# Allantofuranone Biosynthesis and Precursor-Directed Mutasynthesis of Hydroxylated Analogues

**DOI:** 10.1021/acs.jnatprod.5c00197

**Published:** 2025-04-18

**Authors:** Carsten Wieder, Claudia Simon-Sánchez, Johannes C. Liermann, Rainer Wiechert, Karsten Andresen, Eckhard Thines, Till Opatz, Anja Schüffler

**Affiliations:** † Microbiology and Biotechnology, 9182 Johannes Gutenberg University, Hanns-Dieter-Huesch Weg 17, D-55128 Mainz, Germany; ‡ 240097 Institut für Biotechnologie und Wirkstoff-Forschung gGmbH, Hanns-Dieter-Hüsch Weg 17, D-55128 Mainz, Germany; § Department of Chemistry, 9182 Johannes Gutenberg University, Duesbergweg 10-14, D-55128 Mainz, Germany

## Abstract

Genome mining and heterologous reconstitution of biosynthetic genes in Aspergillus oryzae enabled elucidation of the hitherto elusive biosynthetic route that produces allantofuranone (**1**), a bioactive natural product originally isolated from Allantophomopsis lycopodina. The core non-ribosomal peptide synthetase (NRPS)-like enzyme AlfA of the *alf* BGC produces polyporic acid (**2**) from phenylpyruvic acid. In subsequent reactions, compound **2** is reductively dehydrated by the bifunctional enzyme AlfC and methylated by AlfD to produce terferol (**6**). In a final step, the quinol moiety of compound **6** is oxidatively cleaved and contracted by the aromatic ring cleavage dioxygenase AlfB. Using combinatorial biosynthesis, we were able to manipulate the biosynthetic route to yield hydroxylated pathway congeners, most notably the new natural products deoxyascocorynin (**10**), hydroxyterferol (**11**), and hydroxyallantofuranone (**12**).

## Graphical abstract

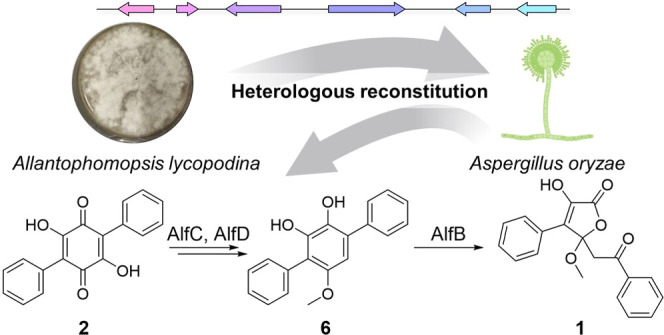

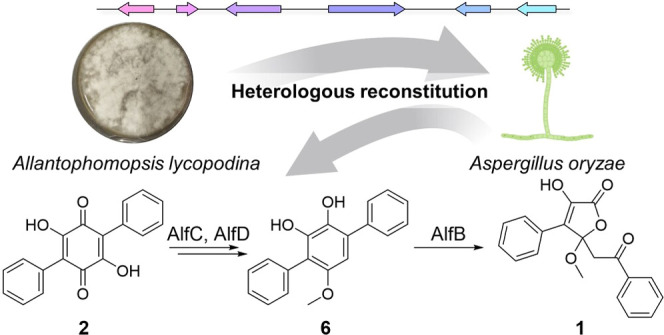

Non-ribosomal peptide synthetases (NRPSs) are multimodular multidomain enzymes that catalyze the ribosome-independent assembly of peptides from proteinogenic and non-proteinogenic amino acids as well as some other keto, hydroxy, and fatty acids.^1^ Canonical NRPS modules are composed of at least an adenylation (A), thiolation (T), and condensation (C) domain that catalyze substrate activation, tethering and peptide bond formation of adjacent T-domain-bound substrates, respectively.^1^ In contrast to canonical NRPSs, NRPS-like enzymes lack a C-domain and can be distinguished into reducing and non-reducing types, harboring either a terminal reductase (R) or thioesterase (TE) domain, respectively.^2,3^ Reducing NRPS-like enzymes are often involved in the reductive tailoring of natural products as in, e.g., the biosynthesis of ascofuranone,^4^ but can also produce metabolites of their own as is the case in the biosynthesis of aspergillic and neoaspergillic acid.^5^ Non-reducing NRPS-like enzymes catalyze the condensation of two identical aromatic α-keto acids, facilitated by the terminal TE domain, to form various different cyclic core structures, i.e., benzoquinones,^6–10^ furanones^11–13^ and dioxolanones^14,15^ (Figure 1). To this date, no non-reducing type NRPS-like enzyme has been reported that deviates from the aforementioned substrate scope, which limits the diversity of resulting products. Instead, diversification of NRPS-like enzyme derived metabolites is achieved through downstream modifications introduced by tailoring enzymes.

**Figure 1:**
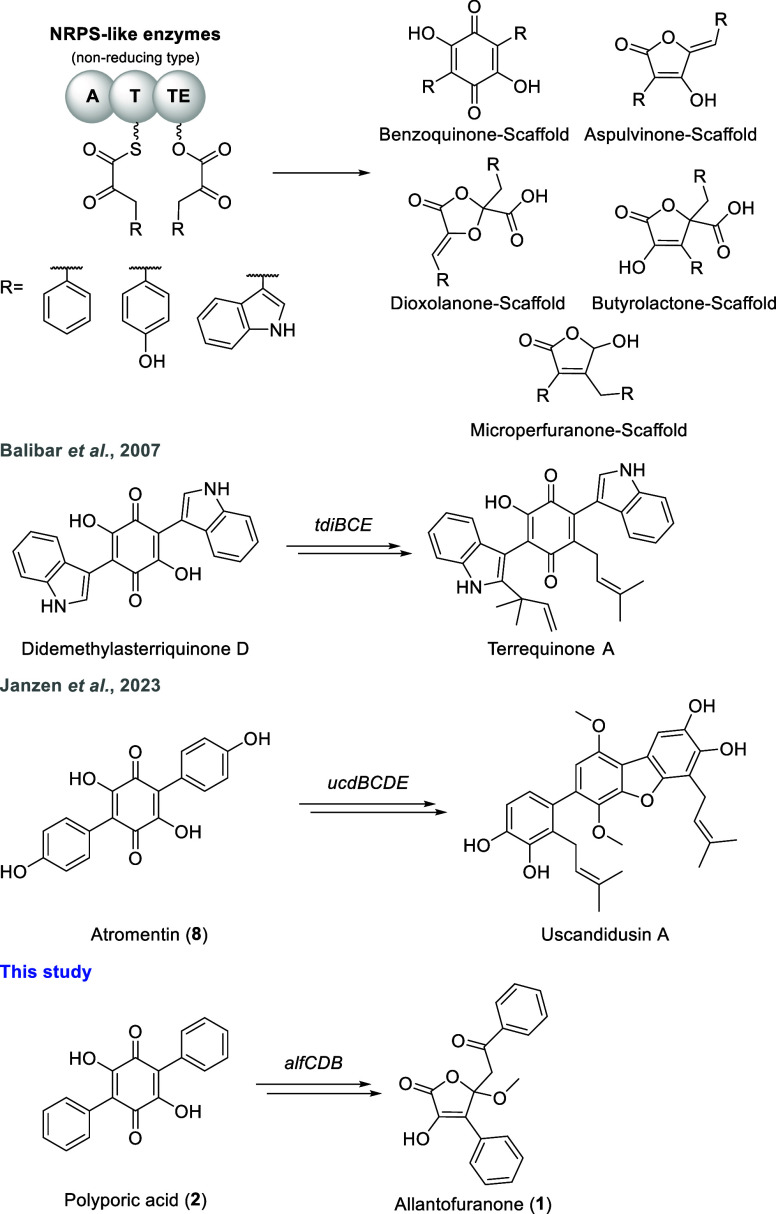
NRPS-like enzyme derived natural products. NRPS-like enzymes catalyze the condensation of two identical aromatic α-keto acids to afford various cyclic products. Increased product diversity is achieved by subsequent tailoring as exemplified by the diversification of benzoquinones.

The biosynthesis of terrequinone A, the first natural product reported to derive from an NRPS-like enzyme, was characterized in 2007 by Balibar et al. through *in vitro* reconstitution of the biosynthetic enzymes^6^ (Figure 1). The NRPS-like enzyme TdiA produces the benzoquinone didemethylasterriquinone D from two molecules of indolepyruvate, which are provided for the reaction by the l-tryptophan aminotransferase TdiD. Next, the quinone reductase TdiC reduces the quinone core which is subsequently prenylated twice by the prenyltransferase TdiB. While the exact function of TdiE is not quite clear, it is required for formation of the diprenylated product and preventing formation of a mono-*O*-prenylated shunt product.

More recently, Janzen et al. characterized the biosynthesis of the dibenzofurans uscandidusin A/B by first introducing the entire *ucd* BGC into the heterologous host Aspergillus nidulans and subsequently deleting biosynthetic genes to study their function and isolate biosynthetic intermediates^16^ (Figure 1). The *ucd* BGC encodes an aminotransferase UcdG that is proposed to provide 4-hydroxyphenylpyruvate from l-tyrosine for the NRPS-like enzyme UcdA which produces the benzoquinone atromentin (**8**). Next, the quinone core is proposed to be reductively dehydrated by a bifunctional enzyme UcdB resulting in formation of a 2,3,5-trihydroxyterphenyl intermediate, however, no biosynthetic intermediate could be observed. This proposed intermediate is subsequently dimethylated, prenylated and hydroxylated in a series of reactions catalyzed by UcdC, UcdD and UcdE to give rise to the intermediates usterphenyllins A/B. These then undergo spontaneous dibenzofuran formation to yield the final products, which seems to be dependent on the *m*-hydroxylation of the phenyl rings.

Furthermore, multiple other NRPS-like biosynthetic pathways have been elucidated including the biosynthesis of aspulvinone H,^12^ butyrolactone I^3,12^ and ascocorynin.^10^ On the other hand, many further natural products have been proposed to derive from NRPS-like enzyme pathways, but their biosynthesis has remained enigmatic as is the case for involutin,^17^ thelephoric acid,^18^ variegatic acid^19^ and guignardic acid.^20^ It is noteworthy, that the physiology of the host can play a crucial role in the production of NRPS-like enzyme derived products, as exemplified by the enzymatic or non-enzymatic modification of benzoquinones in Aspergillus niger^7^ and A. nidulans.^9^

Allantofuranone (**1**) is produced by Allantophomopsis lycopodina and was first reported in 2009.^21^ It was initially isolated because of its moderate antifungal activity against some fungal species. Structurally, compound **1** resembles butyrolactone IIa, however it is differently substituted at the C5 position. In a previous study, the biosynthesis of compound **1** was investigated by means of ^13^C-labeling and feeding of a difluorinated precursor, which hinted toward compound **1** originating from the benzoquinone polyporic acid (**2**)^22^ (Figure 1), which, similar to didemethylasterriquinone D and atromentin (**8**), is also the product of NRPS-like enzymes such as AcyN and CorA.^10,18^ Therefore, the furanone moiety in compound **1** is proposedly produced via post-synthesis ring contraction which is in contrast to the direct furanone formation in butyrolactone IIa and aspulvinone E. The genetic basis of allantofuranone (**1**) biosynthesis has so far been elusive and especially the unique ring contraction sparked our interest. To our knowledge, no enzymes involved in the ring contraction of NRPS-like enzyme derived natural products have been reported to date.

Here, we report the identification of the BGC responsible for allantofuranone (**1**) biosynthesis in A. lycopodina. Heterologous reconstitution of biosynthetic genes in Aspergillus oryzae OP12 allowed for the elucidation of the biosynthetic pathway, which involves dioxygenase-catalyzed ring contraction to produce the furanone moiety found in compound **1**. By employing precursor-directed combinatorial mutasynthesis, it was furthermore possible to produce a new hydroxylated analogue of compound **1** and other mono- and dihydroxylated pathway intermediates.

## Results and Discussion

### Identification of a Candidate Biosynthetic Gene Cluster

The genome of A. lycopodina was sequenced in order to investigate the biosynthetic origin of allantofuranone (**1**). antiSMASH^23^ analysis revealed two biosynthetic gene clusters containing non-reducing NRPS-like enzymes, one of which was investigated due to the predicted functions of adjacent genes. Besides the NRPS-like enzyme *alfA*, the *alf* cluster (accession number PQ256815) encodes a 3-deoxy-d-arabinoheptulosonate-7-phosphate (DAHP)-synthase (*alfS*), an aromatic ring cleavage dioxygenase (*alfB*), a zinc-binding transcription factor (*alfR*), a dehydrogenase (*alfC*), and an *O*-methyltransferase (*alfD*) (Table 1 and Figure 2).

**Table 1:** Proposed Function of *alf* Cluster Genes

gene	size (aa)	BlastP hit^a^	identity (%)	*E* value	proposed protein function
*alfS*	387	C9K7C8.1	54.25	1 × 10^–140^	DAHP synthase
*alfB*	278	A0A0F7CUE8.1	36.27	3.00 × 10^–45^	aromatic ring cleavage dioxygenase
*alfR*	519	B8N0F0.1	25.71	2.00 × 10^–30^	C6-TF
*alfA*	930	P9WES4.1	69.58	0.0	NRPS-like enzyme (A-T-TE)
*alfC*	306	P63936.1	24.26	3.00 × 10^–7^	dehydrogenase
*alfD*	433	Q0CS95.1	41.96	3.00 × 10^–117^	*O*-methyltransferase

a Uniprot as reference database, manually curated choice (best, characterized fungal hit if possible).

**Figure 2:**
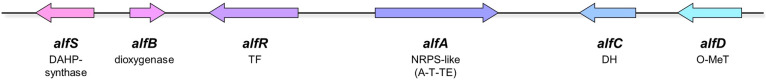
Scheme of *alf* cluster. A, adenylation domain; DAHP, 3-deoxy-d-arabinoheptulosonate-7-phosphate; DH, dehydrogenase; O-MeT, *O*-methyltransferase; T, thiolation domain; TE, thioesterase domain; and TF, transcription factor.

### Elucidation of Allantofuranone Biosynthesis

In order to elucidate the biosynthesis of allantofuranone (**1**), genes encoded in the *alf* cluster were sequentially reconstituted in the heterologous host Aspergillus oryzae OP12.^7^ The resulting mutant strains were analyzed for the production of metabolites absent from the empty plasmid control strain and their respective parental strains (Figure 3). Newly produced metabolites were purified for structure elucidation.

**Figure 3:**
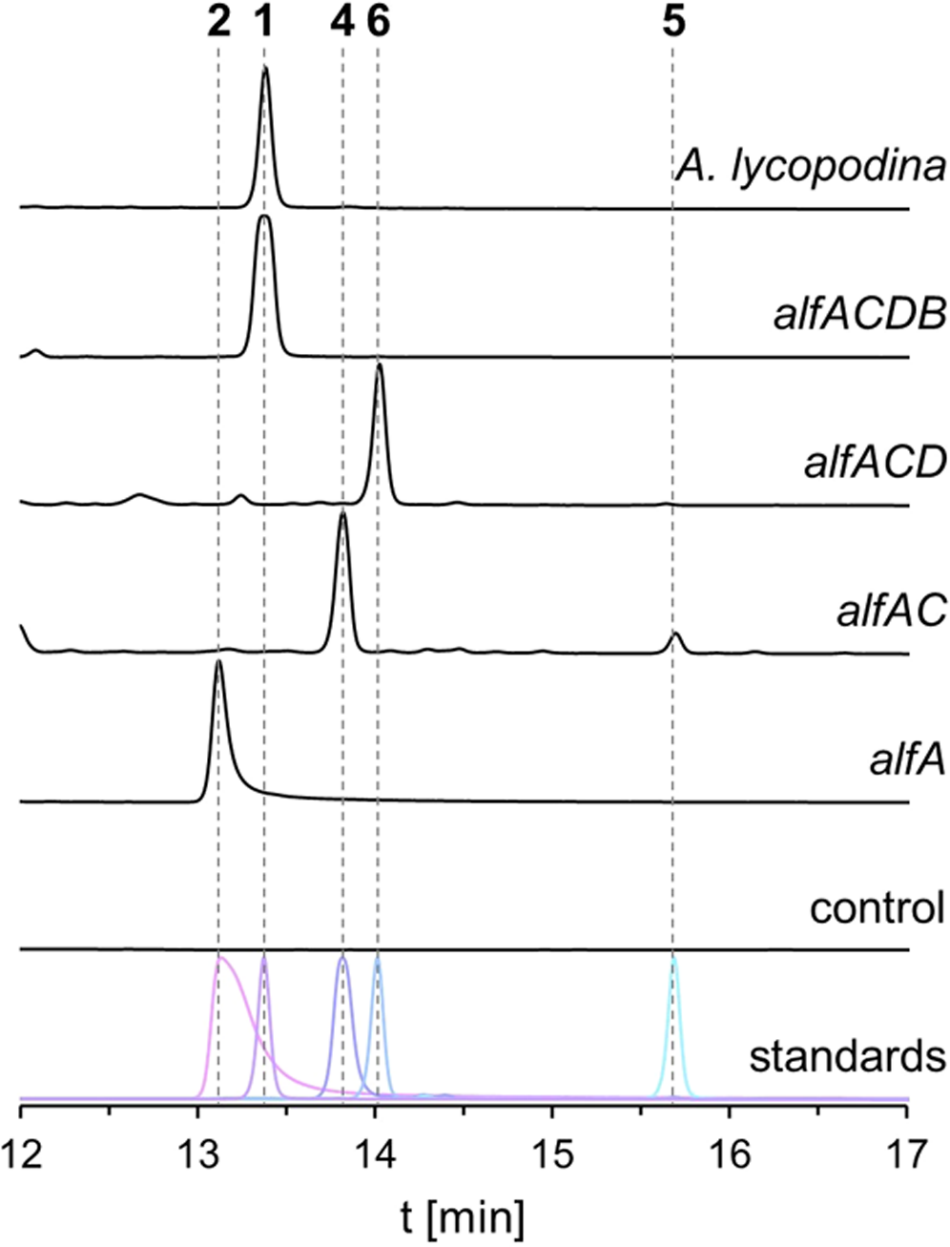
Heterologous reconstitution of allantofuranone (**1**) biosynthesis in A. oryzae OP12. Chromatograms (250 nm) of culture filtrate extracts of OP12 mutant strains expressing *alf* genes, A. lycopodina, and standards. Control, OP12 transformed with empty plasmid.

Heterologous expression of solely the NRPS-like encoding gene *alfA* resulted in the production of polyporic acid (**2**), the presumed precursor of allantofuranone (**1**),^22^ reinforcing the hypothesis, that the *alf* cluster is indeed involved in the biosynthesis of allantofuranone (**1**). Next, coexpression of *alfA* and the predicted dehydrogenase gene *alfC* resulted in formation of the major product deoxypolyporic acid (**4**) and the 5,5-linked symmetric deoxypolyporic acid dimer (**5**), which has not been reported in literature before. We hypothesized that production of compound **4** likely proceeds via the unstable 2,3,5-trihydroxyterphenyl intermediate **3**, which either reoxidizes or dimerizes in the presence of O_2_ (Figure S.4). Indeed, reduction and dehydration of compound **2** to compound **3** as well as the spontaneous reoxidation of compound **3** to compound **4** has previously been reported in the biosynthesis of bacterial echosides, where these reactions are catalyzed by two distinct but collaborating enzymes^24,25^ (Figure S.5). By comparison, *alfC* combines both these functionalities, catalyzing the reductive dehydration of compound **2** to compound **3**. Analogously, reductive dehydration of the related benzoquinone atromentin (**8**) was previously proposed in the biosynthesis of uscandidusins catalyzed by UcdB (accession number KIA75357.1; Figure S.5),^16^ which shares 41.16% homology with AlfC (*E* value: 3.00 × 10^–76^).

Additional coexpression of the *O*-methyltransferase coding gene *alfD* alongside *alfAC* resulted in the production of terferol (**6**), an *O*-methylated derivative of the proposed intermediate **3**. The methylation seems to stabilize the reactive *p*-terphenyl core, as apparent by the absence of dimeric shunt products. Lastly, additional coexpression of the aromatic ring cleavage dioxygenase encoding gene *alfB* resulted in the production of allantofuranone (**1**), which implies the oxidative cleavage of compound **6** and subsequent rearrangement of the linear intermediate into the furanone scaffold. Based on these findings the biosynthetic pathway of compound **1** is proposed as depicted in Scheme 1.

**Scheme 1:**
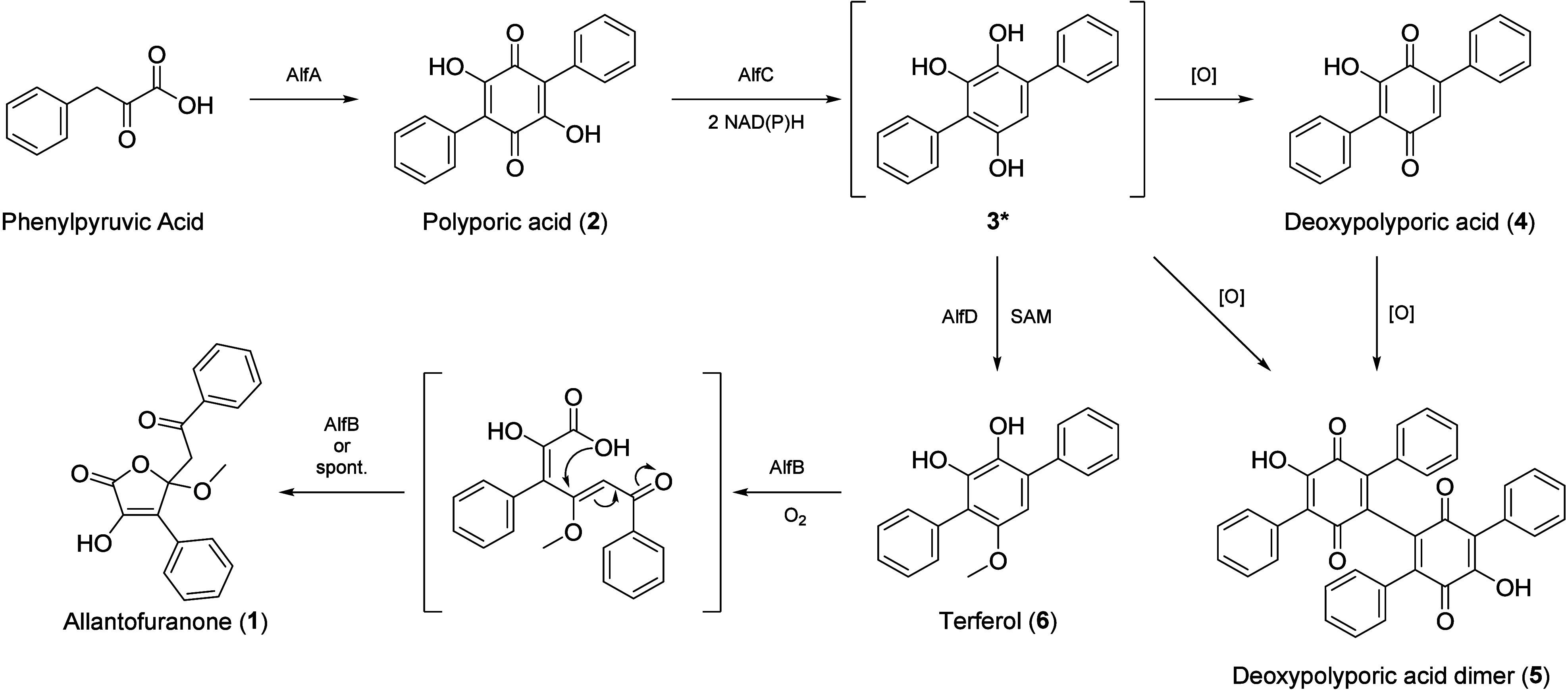
Proposed Biosynthetic Pathway for Allantofuranone (**1**) in A. lycopodina

* Product not observed, structure proposed.

Aromatic ring cleavage dioxygenases are frequently encountered in catabolic pathways for the degradation of aromatic compounds,^26,27^ but to our knowledge have not been reported in natural product biosynthesis as of yet. These enzymes catalyze the ring fission of catecholic substrates by cleaving the aromatic ring either *ortho* (intradiol dioxygenases) or *meta* (extradiol dioxygenases) to the hydroxyl functionalities^26,27^ (Figure S.6). Based on the cleavage pattern AlfB can be categorized as an extradiol-dioxygenase and the cleavage of compound **6** can be compared to the cleavage of polychlorinated-biphenyls by the extradiol-dioxygenase BphC from Pseudomonas sp.^28^ (Figure S.6).

In the β-ketoadipate pathway the intradiol-cleavage of catecholic substrates results in formation of linear muconic acid intermediates, which are subsequently lactonized by cycloisomerases prior to further degradation.^27,29^ These muconolactones structurally resemble the scaffold of allantofuranone (**1**) (Figure S.6) and indeed the muconolactone moiety can be found in a variety of other natural products as well, such as pochoniolides,^30^ terphyl (di-) acid^31^ and terphenolide.^32^ Therefore, the adoption of aromatic ring cleavage dioxygenases into secondary metabolism does not seem to be unique to allantofuranone (**1)** biosynthesis. Whether or not the lactonization of compound **1** from the proposed linear intermediate occurs spontaneously or is favored by AlfB remains elusive. Notably, muconolactones can be spontaneously formed from 2- or 4-alkyl-substituted phenols via their respective muconic acid intermediates when phenols are oxidatively degraded with H_2_O_2_ (Figure S.6).^33^

An interesting aspect of the allantofuranone (**1**) biosynthetic pathway is the high reactivity of the biosynthetic intermediate **3**, resulting in the formation of various dimers in the heterologous host. Similarly, this likely also gives rise to the hybrid terphenyl-naphthalene pigments reportedly produced by A. lycopodina.^34^ This exemplifies a novel type of fungal pigment that is based on the product of an NRPS-like enzyme. While many fungi produce pigments that are based on polymerization of DHN, YWA1, or l-DOPA, Aspergillus terreus has previously also been reported to produce a non-canonical conidial pigment derived through activation and polymerization of the NRPS-like enzyme product aspulvinone E.^11^ Therefore, the biosynthetic pathway of allantofuranone (**1**) might serve an additional purpose in A. lycopodina i.e., protection against ultraviolet (UV) light through production of off-pathway hybrid pigments.

Lastly, it is noteworthy that the isolated yield of compound **1** from OP12_*alfACDB* (110 mg/1 L) exceeded the isolated yield from the natural producer previously reported (191.3 mg/20 L) by 11-fold,^21^ showcasing the power of heterologous expression for natural product synthesis.

### Combinatorial Mutasynthesis of Allantofuranone Analogues

In previous studies it was demonstrated, that the diversity of NRPS-like enzyme derived products can be expanded by employing combinatorial biosynthesis.^3,15,35^ Inspired by these approaches, we attempted to exploit the *alf* biosynthetic genes for precursor-directed mutasynthesis to yield new natural products (Figures 4 and Scheme 2). Since it was previously shown that difluorinated compound **2** can be converted into difluorinated compound **1**,^22^ we hypothesized that the tailoring enzymes downstream of AlfA might also accept ascocorynin (**7**) and atromentin (**8**), mono- and dihydroxylated congeners of compound **2**, resulting in the formation of hydroxylated analogues of the natural pathway intermediates. To this end, the polyporic acid monooxygenase coding gene *AsMO6277* from Ascocoryne sarcoides, which was previously reported to convert compound **2** to compound **7**,^10^ was introduced into all previously established OP12_*alf* mutants. Additionally, OP12 mutants harboring the atromentin (**8**) synthetase *atrA* ^36^ from A. terreus instead of *alfA* alongside the other *alf* genes were constructed. Again, all resulting mutant strains were analyzed for the production of metabolites and new products were purified for structure elucidation.

**Figure 4:**
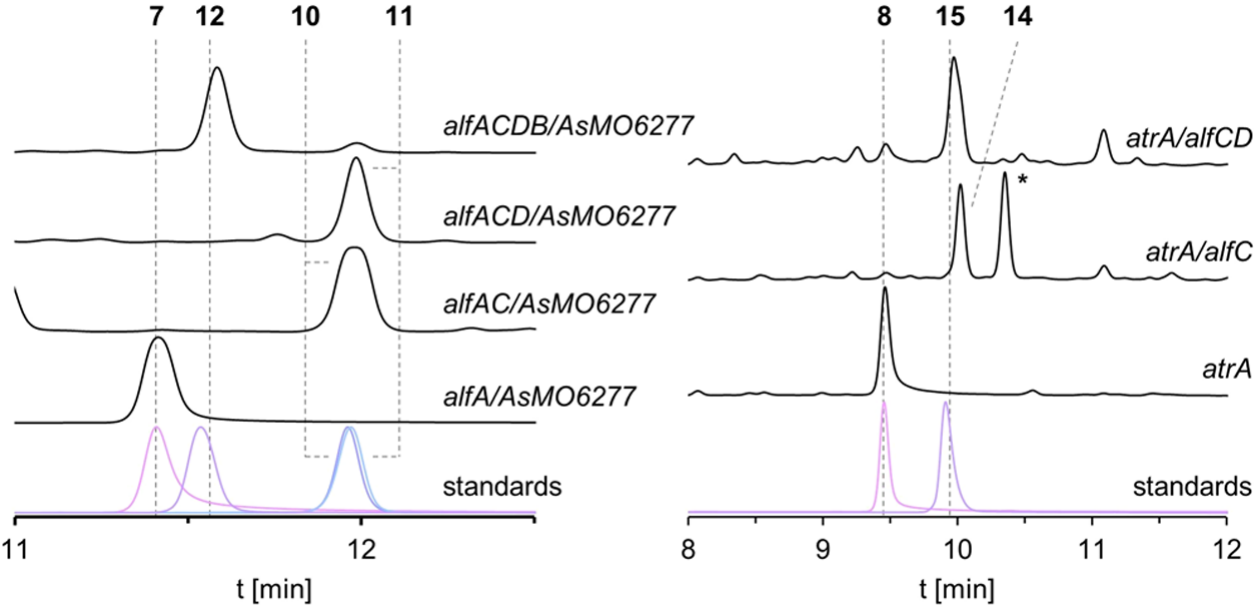
Combinatorial biosynthesis of hydroxylated allantofuranone (**1**) analogues in A. oryzae OP12. Chromatograms (250 nm) of culture filtrate extracts of OP12 mutant strains expressing *alf* genes and either *AsMO6277* or *atrA* and standards. (∗) Uncharacterized dimer.

**Scheme 2:**
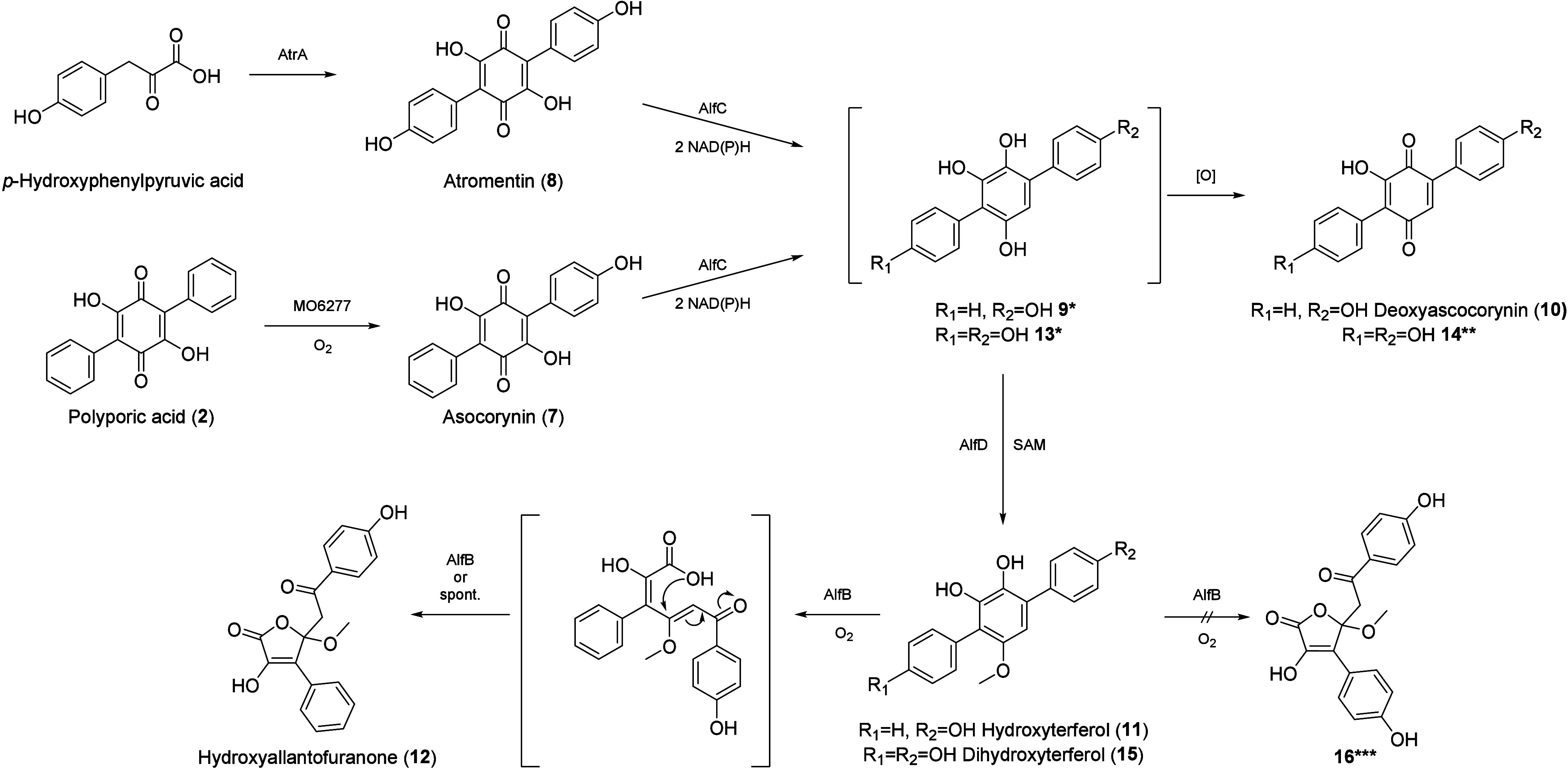
Proposed Mutasynthetic Pathway for Hydroxylated Allantofuranone (**1**) Analogues

* Product not observed, structure proposed.
** Product observed, structure proposed.
*** Hypothetical product, not produced, structure proposed.

As already previously reported, the coexpression of *alfA* and *AsMO6277* in OP12 led to the production of ascocorynin (**7**).^10^ Coexpression of *AsMO6277* alongside *alfAC* led to the production of the new natural product deoxyascocorynin (**10**), which is a monohydroxylated congener of compound **4**. Interestingly, only one position isomer of compound **10** is produced, suggesting AlfC either preferring or only accepting one orientation of substrate **7**. Production of compound **10** is proposed to proceed via the unstable intermediate **9** and similar to OP12_*alfAC*, OP12_*alfAC*/*AsMO6277* also produced dimers. The mutant strain OP12_*alfACD*/*AsMO6277* produced the new natural product hydroxyterferol (**11**), which as expected is a monohydroxylated analogue of compound **6**. Finally, additional coexpression of *alfB* resulted in the production of the new monohydroxylated allantofuranone analogue hydroxyallantofuranone (**12**). The allantofuranone (**1**) biosynthetic machinery was shown to be promiscuous enough to accept ascocorynin (**7**), a monohydroxylated analogue of the first biosynthetic intermediate polyporic acid (**2**), which allowed for the production of the new-to-nature natural products **10**, **11**, and **12** (Scheme 2).

Next, we reconfirmed that expression of solely *atrA* in OP12 did result in the production of atromentin (**8**) as previously reported.^36^ Coexpression of *atrA* with *alfC* resulted in the production of compound **14**. Unfortunately, despite multiple attempts, we were not able to purify compound **14** for structure elucidation, as it was extremely unstable, decomposing and/or dimerizing during the extraction/purification workflow. The identity of compound **14** is proposed as the dihydroxylated congener of compound **4**, which is in line with the detected mass of 307 Da [M – H^+^] and the similarity of the ultraviolet/visible (UV/vis) spectra of compounds **14** and **10** (Figure S.1).

Successful conversion of compound **8** to compound **14** was unexpected, as previously only one position isomer of compound **10** was produced from compound **7** by AlfC. Therefore, while the hydroxyl moieties in either position do not seem to hinder conversion, AlfC might have a stronger affinity toward one substrate orientation when presented with compound **7**. Again, we propose production of compound **14** to proceed via oxidation of intermediate **13**, which seems to be even more unstable compared to proposed intermediates**3** and **9**. Interestingly, while the biosynthesis of uscandidusins A/B is proposed to progress via intermediate **13**, production of compound **14** was not observed in the heterologous host A. nidulans.^16^ This might be due to the high reactivity of compound **13**, which in the presence of other molecules might result in the formation of insoluble or undetectable conjugates in analogy to the formation of terphenyl-naphthalene hybrid pigments in A. lycopodina discussed earlier.

Additional coexpression of *alfD* along *atrA* and *alfC* did successfully result in the production of dihydroxyterferol (**15**), a compound which had previously only been produced synthetically^37^ but not described as a natural product. Unfortunately, OP12_*atrA/alfCDB* did not produce a dihydroxylated analogue (**16**) of allantofuranone (**1**) (Figure S.7), therefore the substrate promiscuity of AlfB seems to be limited. The discrepancy between conversion of monohydroxylated intermediate **11** but non-conversion of dihydroxylated intermediate **15** was unexpected, as difluorinated compound **2** was previously reported to be converted to difluorinated compound **1**.^22^ However, oxygen is both bigger in size and more prone to polar interactions, which might interfere with the catalytic activity of AlfB. This bottleneck could be overcome in the future by additionally employing enzyme engineering. Similarly, engineering of AlfC might allow for accessing the other position isomers of compounds **10**, **11**, and **12**.

Notably, the yield of atromentin (**8**) derived analogues were far lower as compared to the natural allantofuranone (**1**) pathway intermediates. This is likely due to a decreased availability of the substrate 4-hydroxyphenylpyruvic acid as compared to phenylpyruvic acid in the heterologous host. In future efforts this limitation could be overcome by additionally coexpressing a tyrosine transaminase such as *ucdG*. Indeed, deletion of *ucdG* from the uscandidusin BGC in the heterologous host A. nidulans led to a slight decrease in metabolite production.^16^ Multiple other NRPS-like BGCs have been reported to encode specific transaminases for providing α-keto acids to the NRPS-like enzymes such as TdiD in the terrequinone A BGC of A. nidulans^6^ and AtrD in the atromentin (**8**) BGC of Tapinella panuoides.^8^

Lastly, there are numerous other benzoquinone and terphenyl natural products that contain unique modifications, the biosynthetic origin of which have not been characterized as of yet, but once elucidated can potentially also be harnessed for combinatorial biosynthesis in the future. Among others, these include various methylations, hydroxylations, *C*- and *O*-prenylations, cyclized prenyl moieties and the previously mentioned proximal muconolactone moieties.^38–47^

### Structure Elucidation of Purified Compounds

All purifed compounds have been characterized using one-dimensional (1D) and two-dimensional (2D) nuclear magnetic resonance (NMR) as well high-resolution electrospray ionization mass spectrometry (HRESIMS). Allantofuranone (**1**) and six more compounds (**2**, **4**, **6**–**8**, and **15**) have previously been described and their analytical data is in accordance with the literature.

Deoxypolyporic acid dimer (**5**) was found to have a molecular formula of C_36_H_22_O_6_ by HRESIMS. The NMR spectra were very similar to those of deoxypolyporic acid (**4**). The molecular formula and the high similarity indicated a symmetrical homodimer of compound **4**. This was confirmed by the lacking of the quinone methine group (δ_H_ 6.88, δ_C_ 133.4) and an additional quaternary carbon atom (δ_C_ 140.0). Deoxyascocorynin (**10**) had an elemental formula of C_18_H_12_O_4_ according to HRESIMS. Again, the NMR spectra showed similarity to those of compound **4**. The molecular formula indicated an additional hydroxyl group and one phenyl residue gave an AA′BB′ spin system. Thus, it could be concluded that one of the phenyl residues was *p*-hydroxylated. Characteristic ^3^ *J* heteronuclear multiple bond correlations (HMBCs) (6.78 → 123.1, 7.47 → 141.7) and an nuclear Overhauser effect (NOE) (6.78 ↔ 7.47) showed that the aryl residue was facing the quinone methine group. Hydroxyterferol (**11**, C_19_H_16_O_4_) was analyzed analogously to compound **10**. The central ring of the terphenyl scaffold could be exhaustingly characterized by HMBC correlations from 3-OH (8.17 → 117.0, 144.9, 136.1), 4-OH (7.91 → 144.9, 136.1, 127.8), and 1-OMe (3.59 → 150.3). Again,^3^ *J* HMBC correlations (6.35 → 129.5) and NOE (6.35 ↔ 7.42) proved a substitution pattern corresponding to compound **10**. Hydroxyallantofuranone (**12**) had an elemental formula of C_19_H_16_O_6_ according to HRESIMS. The spectra showed high similarity to those of allantofuranone (**1**) with one phenyl residue again giving an AA′BB′ spin system. Together with the molecular formula, it could be concluded again that one phenyl residue was *p*-hydroxylated. Its location was determined by HMBC to be connected to the ketone (7.72 → 192.3) while the other phenyl residue showed a correlation into the furanone ring (7.83 → 121.3).

### Biological Activity of Purified Compounds

The antimicrobial activity of the purified compounds was assessed in routine bioassays, covering germination inhibition of filamentous ascomycetes including various plant pathogenic species, growth inhibition of dimorphic human pathogenic yeast Candida albicans, potato blight oomycete Phytophthora infestans and some bacterial strains including human pathogenic Staphylococcus aureus and Pseudomonas aeruginosa (Table 2). Ciclopirox (100 μg/mL) was used as an experiment positive control, fully inhibiting germination and growth of all tested fungi and P. infestans. Streptomycin (100 μg/mL) and tetracycline (100 μg/mL) were used as experiment positive controls, fully inhibiting growth of all tested bacteria. Apart from compound **12**, all compounds exhibited some, mostly mild bioactivity in the performed assays. The most noteworthy activities include the anti-Phytophthora activity of compound **4** at an MIC of 5 μg/mL, the germination inhibitory activity of compound **6** against Fusarium graminearum at a MIC of 10 μg/mL and the anti-Candida and anti-Phytophthora activity of compound **10** with MICs of 10 and 5 μg/mL, respectively. However, as these compounds are not only active against one species, but broadly active instead (even across domains), none of the purified compounds poses a valuable drug lead. Interestingly, comparing compounds **4** and **10**, hydroxylation did improve activity in some assays up to 10-fold (antifungal activity against C. albicans) and decreased activity in others, showcasing the effect of even minor molecular changes on bioactivity.

**Table 2:** Antimicrobial Activity of Purified Compounds^e^

	MIC (μg/mL)										
organism	**1**	**2**	**4**	**5**	**6**	**7**	**8**	**10**	**11**	**12**	**15**
Magnaporthe oryzae (H_2_O)^a^	50	–	–	50	5	50	–	50	50	–	–
Magnaporthe oryzae (CM)^a^	50	50	>100	50	10	50	–	10	50	–	–
Botrytis cinerea ^a^	>100^c^	>100	>100	–	100	–	–	10	100	–	>100
Fusarium graminearum ^a^	–^d^	–	–	–	10	–	–	50	50	–	–
Aspergillus oryzae ^a^	>100	–	–	–	–	–	–	100	–	–	–
Candida albicans ^b^	–	–	>100	–	100	–	–	10	100	–	–
Phytophthora infestans ^b^	>100	100	5	–	50	–	–	5	50	–	–
Staphylococcus aureus ^b^	–	50	–	–	50	–	–	100	50	–	100
Pseudomonas aeruginosa ^b^	–	–	–	–	–	–	–	–	–	–	–
Aneurinibacillus migulanus ^b^	–	–	10	50	50	–	–	50	50	–	50
Enterobacter cloacae subsp. dissolvens ^a^	–	–	–	–	–	–	–	–	–	–	–

a Ciclopirox (100 μg/mL) was used as a positive control, fully inhibiting germination and growth of all tested fungi and oomycetes. Streptomycin (100 μg/mL) and tetracycline (100 μg/mL) were used as positive controls (separately), fully inhibiting the growth of all tested bacteria.

b Inhibition of conidial germination.

c Inhibition of growth.

d Partially inhibited at maximum test concentration.

e No activity at 100 μg/mL.

## Summary

In summary, the allantofuranone (**1**) biosynthetic gene cluster was identified in A. lycopodina through genome mining and biosynthesis was elucidated through heterologous reconstitution in A. oryzae OP12. Our results confirm the previous finding that the biosynthesis of compound **1** progresses via polyporic acid (**2**) as the first intermediate. The bifunctional enzyme AlfC catalyzes benzoquinone to *p*-terphenyl conversion through reductive dehydration. The unstable intermediate **3** either spontaneously reoxidizes to compound **4**, dimerizes to compound **5**, reacts with naphthalene-compounds to form a novel type of hybrid pigment, or is stabilized through O-methylation by AlfD. In a final reaction, AlfB oxidatively cleaves the *p*-terphenyl core of intermediate **6** which is subsequently rearranged to afford the final furanone scaffold in compound **1**. Additionally, we report combinatorial mutasynthesis of new hydroxylated analogues of natural pathway intermediates (**10**, **11**, and **12**), highlighting the potential of engineering biosynthetic pathways in accessing non-natural chemical diversity.

## Experimental Section

### General Experimental Procedures

Optical rotation measurements were accomplished with a PerkinElmer 241MC polarimeter at λ = 589 nm. A solvent-filled cuvette was used for instrument calibration.^48^ UV/vis spectra of compounds were extracted from high-performance liquid chromatography (HPLC) runs (Figure S.1). Infrared spectroscopy was performed on a Bruker Tensor 27 FTIR spectrometer including a diamond ATR unit and is reported in terms of absorption frequency *v̅* (cm^–1^). NMR spectra were recorded at 294 K on a 600 MHz Bruker Avance-III 600 spectrometer equipped with a 5 mm TCI cryoprobe. ^1^H and ^13^C chemical shifts are given relative to tetramethylsilane (TMS). ^1^H shifts were calibrated using the residual solvent signal (CDCl_3_: 7.26 ppm; DMSO-*d*_6_: 2.50 ppm).^49^ ^13^C shifts were calibrated using absolute reference from the ^1^H spectra. HRMS was conducted on an Agilent G6545A Q-ToF with ESI, APCI or APPI source coupled with an Agilent 1260 Infinity II HPLC system. For analytical thin-layer chromatography (TLC) 0.25 mm silica plates (60 F254) from Merck were used, and the detection was reached by fluorescence quenching under UV light (λ = 254 nm) or by staining with potassium permanganate reagent (solution of KMnO_4_ (3 g), K_2_CO_3_ (20 g), 5% NaOH (5 mL), and H_2_O (300 mL)) followed by heating at 400 °C. HPLC–MS analysis was performed using a LiChrospher 100 RP-18 column (125 × 2 mm, 4 μm, Merck KGaA) attached to an Agilent DAD 1260 module and a Quadrupole LC/MS 6130 module. For analytical runs, 0.1% formic acid in H_2_O and acetonitrile (ACN) were used as eluents, running a gradient from 1% to 100% ACN in 20 min followed by 100% ACN for 4 min at 0.4 mL/min flow before re-equilibrating. Preparative HPLC was performed using a Sunfire C18 column (100 Å, 5 μm, 19 × 250 mm, Waters GmbH) running on isocratic flow using 0.1% formic acid in H_2_O and ACN as eluents at 17 mL/min flow.

### Fungal Strains and Cultivation Conditions

A. lycopodina IBWF58B-05A and A. oryzae OP12 were routinely cultivated on YMG (0.4% yeast extract, 1% malt extract, and 1% glucose at pH 5.5) and GG10 (50 mM glucose, 10 mM glutamine, 0.52 g/L KCl, 0.52 g/L MgSO_4_·7H_2_O, and 1.52 g/L KH_2_PO_4_; 1 mL/L Hutner’s trace elements; pH 6.5), respectively. Media for auxotrophic mutants were supplemented with 10 mM uridine (OP12 *pyrG* ^–^ and counterselected mutants) or 10 mM uridine, 0.0001% *p*-aminobenzoic acid (PABA) and 0,05% arginine (OP12 3Δ). For induction of expression, OP12 mutant strains were cultivated in 2% starch media (2% soluble starch, 20 mM glutamine, 0.52 g/L KCl, 0.52 g/L MgSO_4_·7H_2_O, and 1.52 g/L KH_2_PO_4_; 1 mL/L Hutner’s trace elements; pH 6.5). All mutant strains used in this study are listed in Table S.1.

### Genome Sequencing and Bioinformatic Analysis

For isolation of genomic DNA, lyophilized mycelium of A. lycopodina was extracted with the GeneJET Plant Genomic DNA Purification Kit (Thermo Scientific) according to the manufacturer’s instructions. Whole genome sequencing was performed by the Institut für Molekulargenetik NGS-Einheit, Mainz, Germany, using a genome sequencer Illumina HiSeq 2500 to generate 5 929 011 paired end reads with a length of 150 nucleotides each. The genome was assembled by using the Software SPAdes^50^ version 3.15.4 to a total length of 39284797 bp in 4383 contigs with an N50 value of 73. Gene prediction was performed by using AUGUSTUS version 3.4.0 ^51^ and resulted in 8752 open reading frames. The set of predicted genes was used in antiSMASH version 6.1.1 ^23^ analysis that revealed 39 BGCs. The *alf* BGC was further analyzed using BLAST^52^ and Interpro.^53^

### Plasmid Construction

Q5 Hot Start High-Fidelity DNA Polymerase (NEB) was used for all PCR amplifications according to the manufacturer’s instructions, PCR products were purified with Monarch PCR & DNA Cleanup Kit (NEB) and plasmids assembled using NEBuilder HiFi DNA Assembly (NEB). Oligonucleotides for all amplification reactions are listed in Table S.2. Coding sequences of *alfA*, *alfB*, *alfC*, *alfD*, *AsMO6277*, and *atrA* were amplified from genomic DNA of A. lycopodina, A. sarcoides DSM 4705, and A. terreus FGSC A1156 and assembled into *Nco*I restricted SM-Xpress_Ura.^7^ Additionally, *alfC* and *alfD* amplicons were assembled into *Nco*I SM-Xpress_paba^10^ and SM-Xpress_argB­(mut),^54^ respectively. The general cloning strategy is schematically depicted in Figure S.2. Escherichia coli DH5α cells (NEB) were used to propagate assembled plasmids. Plasmids were isolated using the Monarch Plasmid Miniprep Kit (NEB) and correct assembly was confirmed by enzymatic restriction.

### Construction of A. oryzae OP12 Mutant Strains

A. oryzae OP12 (*pyrG* ^–^ and 3Δ) protoplast transformations were carried out as previously described.^7,54^ Mutants were constructed by sequentially introducing one plasmid at a time. The URA-cassette in the SM-Xpress_Ura plasmid complements the uridine auxotrophy of OP12, therefore allowing for selection of prototrophic mutants. In between transformations mutant strains were counterselected to reobtain uridine auxotrophy to allow reuse of the same selection marker. Despite multiple efforts, we were unable to counterselect OP12_*atrA*/*alfCD* while maintaining production of compound **15**. Therefore, we reconstructed OP12_*atrA*/*alfCD* in the triple auxotrophic strain OP12 3Δ (*pyrG* ^–^, Δ*pabA*, Δ*argB*)^54^ by simultaneously introducing *atrA*, *alfC*, and *alfD* resulting in strain OP12­(3Δ)_ *atrA*/*alfCD*, which we were able counterselect to subsequently obtain the strain OP12­(3Δ)_ *atrA*/*alfCDB*. Integration of genes was confirmed by diagnostic PCR using the Phire Green Hot Start II PCR Master Mix (Thermo Fisher) (Figure S.3).

### Counterselection

Uridine prototrophic (*pyrG* ^+^) mutants were counterselected on 5-FOA plates (GG10 supplemented with 2 mg/mL FOA, 50 mM HEPES at pH 7.0, and 20 mM uridine) as previously described.^54^ Resulting uridine auxotrophic (*pyrG* ^–^) mutants were then again analyzed for secondary metabolite production before subsequent transformations.

### Fermentation, Extraction, and Metabolite Purification

For screening metabolite production A. oryzae OP12 mutant spores were inoculated into 50 mL 2% starch media and cultivated shaking at 150 rpm for 2 days at 30 °C. Cultures were then filtered over miracloth, and the culture filtrate acidified with HCl (helps with solvent solubility of benzoquinones and terphenyls) before liquid/liquid extraction with an equal amount of ethyl acetate. The organic layer was filtered through anhydrous Na_2_SO_4_ and dried under reduced pressure at 45 °C. Extracts were dissolved in MeOH, centrifuged and applied to HPLC–MS analysis.

For product isolation, mutant spores were first inoculated into 50 mL YEPD media (1% yeast extract, 2% peptone, and 0.5% glucose at pH 6.5) and incubated shaking at 150 rpm overnight at 30 °C. The mycelium was then rinsed with sterile water and transferred to a 1 L 2% starch media main culture and incubated shaking at 120 rpm for another 3 days at 28 °C. Culture filtrate was extracted as previously described. For purification of compound **10**, the media was supplemented with 10 g of HP20 resin to prevent excessive dimerization and decomposition. Instead of extracting the culture filtrate, in this case, the mycelium and resin were extracted instead by submersion in ethyl acetate and shaking for 2 h. Dried extracts were dissolved in DMSO and applied to preparative HPLC. Eluent composition for purification of different compounds is listed alongside pure substance yields in Table 3. Fractions containing the compounds of interest were combined and dried under reduced pressure at 45 °C.

**Table 3:** Eluent Composition Preparative HPLC and Compound Yields

compound	ACN (%)	yield (mg)
**1**	55	110.0
**2**	70	5.2
**4**	55	19.2
**5**	55	17.4
**6**	50	31.3
**7**	45	50.8
**8**	30	14.0
**10**	40	26.2
**11**	40	28.2
**12**	40	54.5
**15**	30	5.3

Allantofuranone (**1**): off-white yellowish amorphous solid; [α]_D_^21^ = +1.3 (c = 0.15, MeOH); *R*_f_ 0.21 (*^c^* Hex/EtOAc 3:1); IR (ATR): *ṽ* [cm^–1^] 2935, 1763, 1681, 1597, 1448, 1388, 1359, 1302, 1177, 1145; HRESIMS *m*/*z* 323.0934 [M – H]^−^ (calcd for [C_19_H_15_O_5_]^−^ 323.0925); ^1^H and ^13^C NMR see Table S.3. The analytical data are in accordance with the literature.^21^

Polyporic acid (**2**): red/brown/bronze amorphous solid; *R*_f_ 0.16 (DCM/MeOH/AcOH 10:1:0.5); IR (ATR) *ṽ* [cm^–1^] 3306, 2916, 2851, 1613, 1595, 1524, 1497, 1399, 1248, 1002; HRESIMS *m*/*z* 291.0668 [M – H]^−^ (calcd for [C_18_H_11_O_4_]^−^ 291.0663); ^1^H and ^13^C NMR, see Table S.5. The analytical data are in accordance with the literature.^55^

Deoxypolyporic acid (**4**): vibrant red powder; *R*_f_ 0.35 (*^c^* Hex/EtOAc 3:1); IR (ATR) *ṽ* [cm^–1^] 3358, 2920, 1665, 1625, 1520, 1493, 1440, 1397, 1116, 1023; HRESIMS *m*/*z* 275.0721 [M – H]^−^ (calcd for [C_18_H_11_O_3_]^−^ 275.0714); ^1^H and ^13^C NMR, see Table S.5. The analytical data are in accordance with the literature.^56^

Deoxypolyporic acid dimer (**5**): red/brown amorphous solid; *R*_f_ 0.13 (*^c^* Hex/EtOAc 3:1); IR (ATR) *ṽ* [cm^–1^] 3366, 2923, 1659, 1526, 1493, 1440, 1369, 1297, 1133, 1019; HRESIMS *m*/*z* 549.1342 [M – H]^−^ (calcd for [C_36_H_21_O_6_]^−^ 549.1344); ^1^H and ^13^C NMR, see Table S.5.

Terferol (**6**): purple oil; *R*_f_ 0.40 (*^c^* Hex/EtOAc 3:1); IR (ATR) *ṽ* [cm^–1^] 3355, 2929, 2851, 1598, 1414, 1371, 1304, 1227, 1106, 1068; HRESIMS *m*/*z* 291.1037 [M – H]^−^ (calcd for [C_19_H_15_O_3_]^−^ 291.1027); ^1^H and ^13^C NMR, see Table S.4. The analytical data are in accordance with the literature.^57^

Ascocorynin (**7**): green/gold powder; *R*_f_ 0.23 (DCM/MeOH/AcOH 10:1:0.5); IR (ATR) *ṽ* [cm^–1^] 3308, 2922, 1610, 1517, 1319, 1310, 1242, 997, 946, 721; HRESIMS *m*/*z* 307.0624 [M – H]^−^ (calcd for [C_18_H_11_O_5_]^−^ 307.0612); ^1^H and ^13^C NMR, see Table S.5. The analytical data are in accordance with the literature.^58^

Atromentin (**8**): red/brown/bronze amorphous solid; *R*_f_ 0.08 (DCM/MeOH/AcOH 10:1:0.5); IR (ATR) *ṽ* [cm^–1^] 2922, 2851, 1606, 1581, 1516, 1437, 1404, 1377, 1236, 1017; HRESIMS (ESI) *m*/*z* 323.0566 [M – H]^−^ (calcd for [C_18_H_11_O_6_]^−^ 323.0561); ^1^H and ^13^C NMR, see Table S.5. The analytical data are in accordance with the literature.^59^

Deoxyascocorynin (**10**): red/brown amorphous solid; *R*_f_ 0.11 (*^c^* Hex/EtOAc 3:1); IR (ATR) *ṽ* [cm^–1^] 3251, 2929, 1660, 1625, 1514, 1494, 1439, 1365, 1347, 1113; HRESIMS *m*/*z* 291.0673 [M – H]^−^ (calcd for [C_18_H_11_O_4_]^−^ 291.0663); ^1^H and ^13^C NMR, see Table S.5.

Hydroxyterferol (**11**): brown/bronze amorphous solid; *R*_f_ 0.11 (*^c^* Hex/EtOAc 3:1); IR (ATR) *ṽ* [cm^–1^] 3313, 1611, 1517, 1412, 1271, 1233, 1065, 1015, 951, 700; HRESIMS *m*/*z* 307.0986 [M – H]^−^ (calcd for [C_19_H_15_O_4_]^−^ 307.0976); ^1^H and ^13^C NMR, see Table S.4.

Hydroxyallantofuranone (**12**): off-white amorphous solid; [α]_D_^21^ = +0.6 (*c* = 0.48, MeOH); *R*_f_ 0.06 (*^c^* Hex/EtOAc 3:1); IR (ATR) *ṽ* [cm^–1^] 2935, 1760, 1688, 1601, 1582, 1287, 1223, 1169, 1016, 951; HRESIMS *m*/*z* 339.0883 [M – H]^−^ (calcd for [C_19_H_15_O_6_]^−^ 339.0874); ^1^H and ^13^C NMR, see Table S.3.

Dihydroxyterferol (**15**): off-white/gray amorphous solid; *R*_f_ 0.33 (*^c^* Hex/EtOAc 1:1); IR (ATR) *ṽ* [cm^–1^] 3220, 1610, 1514, 1437, 1269, 1232, 1173, 1015, 951, 835; HRESIMS *m*/*z* 323.0933 [M – H]^−^ (calcd for [C_19_H_15_O_5_]^−^ 323.0925); ^1^H and ^13^C NMR, see Table S.4. The analytical data are in accordance with the literature.^37^

### Bioactivity Assays

#### Germination Inhibition of Ascomycete Fungi

Conidia of Magnaporthe oryzae 70-15, Botrytis cinerea DSM 0877, Fusarium graminearum DSM 21727, and A. oryzae RIB40 were harvested from properly grown agar plates and diluted in 2% malt extract media to a final concentration of 1 × 10^5^ conidia/mL. A total of 200 μL of the solution were added to wells of a 96-well plate containing different concentrations of compounds. The plates were then incubated overnight at room temperature and conidia germination evaluated using a microscope. Ciclopirox (100 μg/mL) served as positive control.

#### Growth Inhibition of Dimorphic Yeast C. albicans

Fresh colonies of C. albicans ATCC90028, grown on Sabouraud (Difco) plates, were suspended in H_2_O, diluted 1:20 in Sabouraud media, 200 μL distributed in 96-well test plates and cultivated shaking at room temperature for 18–24 h; growth inhibition was assessed macroscopically. Ciclopirox (100 μg/mL) served as positive control.

#### Growth Inhibition of Oomycete P. infenstans

A total of 2 mL of a 2-week-old liquid PDA culture of P. infenstans CBS 430.90 were shredded using a FastPrep twice for 20 s, diluted with 5 mL of H_2_O, and filtered through miracloth. The filtrate was diluted 1:20 with PDB media (Difco) and 200 μL distributed in 96-well test plates. Plates were incubated gently shaking at room temperature for 1 week; growth inhibition was assessed macroscopically. Ciclopirox (100 μg/mL) served as positive control.

#### Growth Inhibition of Bacteria

Nutrient broth (Difco) precultures of Staphylococcus aureus ATCC11632 (37 °C), Pseudomonas aeruginosa ATCC15442 (37 °C), Aneurinibacillus migulanus ATCC9999 (37 °C), and Enterobacter cloacae subsp. dissolvens LMG2683 (27 °C) were grown overnight shaking. Precultures were diluted 1:100 in fresh nutrient broth and 200 μL were distributed in 96-well test plates. Plates were cultivated shaking at 37 or 27 °C for 18–24 h and growth inhibition was assessed macroscopically. Tetracycline (100 μg/mL) and streptomycin (100 μg/mL) served as positive controls.

## Supplementary Material



## Data Availability

All data underlying this study is available in this article and the Supporting Information. The allantofuranone (*alf*) biosynthetic gene cluster has been deposited at NCBI (accession number PQ256815). The analytical data (NMR spectra, MS spectra, and IR spectra) for all purified compounds has been deposited at Chemotion Repository (10.14272/collection/JCL_2025-02-05).

## Notes

Supplementary tables (oligonucleotides and mutant strains), UV/vis spectra of compounds, cloning strategy scheme, mutant strain validation, chromatograms for OP12_*atrA*/*alfCDB*, NMR assignments, and NMR spectra (PDF)
