# Supplementary material for: Allantofuranone Biosynthesis and Precursor-Directed Mutasynthesis of Hydroxylated Analogues
Source: J Nat Prod. 2025 Apr 18;88(5):1191–200. doi: 10.1021/acs.jnatprod.5c00197 (PMC12105029; doi:10.1021/acs.jnatprod.5c00197)
Supplement: Supplementary file 1 [file np5c00197_si_001.pdf]

Supporting Information

Allantofuranone Biosynthesis and Precursor-Directed Mutasynthesis of Hydroxylated Analogues

Carsten Wieder[1,2,*], Claudia Simon-Sánchez[1], Johannes C. Liermann[3], Rainer Wiechert[3], Karsten Andresen[1], Eckhard Thines[1,2], Till Opatz[3], Anja Schüffler[2,*]

[1] Institute of Molecular Physiology, Johannes Gutenberg-University, Hanns-Dieter-Hüsch Weg 17, D-55128 Mainz, Germany

[2] Institut für Biotechnologie und Wirkstoff-Forschung gGmbH, Mainz, Hanns-Dieter-Hüsch Weg 17, D-55128 Mainz, Germany

[3] Department of Chemistry, Johannes Gutenberg-University, Duesbergweg 10–14, D-55128 Mainz, Germany

*Correspondence: cawieder@uni-mainz.de, schueffler@ibwf.de

Table of contents

Supplementary Tables 2

Supplementary Figures 4

NMR Assignments 9

NMR Spectra 12

References 21

Supplementary Tables

Table S. 1: *Aspergillus oryzae* mutant strains used in this study

Strain	Parental Strain	Genotype	Produces	Source
OP12 *pyrG⁻*	See ref.	PamyB:terR_ptrA; *pyrG⁻*	/	[1]
OP12 3Δ	See ref.	PamyB:terR_ptrA; *pyrG⁻*, *ΔpabA*, *ΔargB*	/	[2]
OP12 empty plasmid	OP12 *pyrG⁻*	PamyB:terR_ptrA, *pyrG⁺*	/	This study
OP12 3Δ empty plasmids	OP12 3Δ	PamyB:terR_ptrA, *pyrG⁺*, *pabA⁺*, *argB⁺*	/	[2]
OP12_*alfA*	OP12 *pyrG⁻*	PamyB:terR_ptrA; PterA:alfA_URA	**2**	This study
OP12_*alfA pyrG⁻*	OP12_*alfA*	PamyB:terR_ptrA; PterA:alfA_URA, *pyrG⁻*	**2**	This study
OP12_*alfAC*	OP12_*alfA pyrG⁻*	PamyB:terR_ptrA; PterA:alfA_URA, PterA:alfC_URA	**4, 5**	This study
OP12_*alfAC pyrG⁻*	OP12_*alfAC*	PamyB:terR_ptrA; PterA:alfA_URA, PterA:alfC_URA, *pyrG⁻*	**4, 5**	This study
OP12_*alfACD*	OP12_*alfAC pyrG⁻*	PamyB:terR_ptrA; PterA:alfA_URA, PterA:alfC_URA, PterA:alfD_URA	**6**	This study
OP12_*alfACD pyrG⁻*	OP12_*alfACD*	PamyB:terR_ptrA; PterA:alfA_URA, PterA:alfC_URA, PterA:alfD_URA, *pyrG⁻*	**6**	This study
OP12_*alfACDB*	OP12_*alfACD pyrG⁻*	PamyB:terR_ptrA; PterA:alfA_URA, PterA:alfC_URA, PterA:alfD_URA, PterA:alfB_URA	**1**	This study
OP12_*alfACDB pyrG⁻*	OP12_*alfACDB*	PamyB:terR_ptrA; PterA:alfA_URA, PterA:alfC_URA, PterA:alfD_URA, PterA:alfB_URA, *pyrG⁻*	**1**	This study
OP12_*alfA/AsMO6277*	OP12_*alfA pyrG⁻*	PamyB:terR_ptrA; PterA:alfA_URA, PterA:AsMO6277_URA	**7**	This study
OP12_*alfAC/AsMO6277*	OP12_*alfAC pyrG⁻*	PamyB:terR_ptrA; PterA:alfA_URA, PterA:alfC_URA, PterA:AsMO6277_URA	**10**	This study
OP12_*alfACD/AsMO6277*	OP12_*alfACD pyrG⁻*	PamyB:terR_ptrA; PterA:alfA_URA, PterA:alfC_URA, PterA:alfD_URA, PterA:AsMO6277_URA	**11**	This study
OP12_*alfACDB/AsMO6277*	OP12_*alfACDB pyrG⁻*	PamyB:terR_ptrA; PterA:alfA_URA, PterA:alfC_URA, PterA:alfD_URA, PterA:alfB_URA, PterA:AsMO6277_URA	**12**	This study
OP12_*atrA*	OP12 *pyrG⁻*	PamyB:terR_ptrA; PterA:atrA_URA	**8**	This study
OP12_*atrA pyrG⁻*	OP12_*atrA*	PamyB:terR_ptrA; PterA:atrA_URA, *pyrG⁻*	**8**	This study
OP12_*atrA/alfC*	OP12_*atrA pyrG⁻*	PamyB:terR_ptrA; PterA:atrA_URA, PterA:alfC_URA	**13, 14**	This study
OP12_*atrA/alfC pyrG⁻*	OP12_*atrA/alfC*	PamyB:terR_ptrA; PterA:atrA_URA, PterA:alfC_URA, *pyrG⁻*	**13, 14**	This study
OP12_*atrA/alfCD*	OP12_*atrA/alfC pyrG⁻*	PamyB:terR_ptrA; PterA:atrA_URA, PterA:alfC_URA, PterA:alfD_URA	**15**	This study
OP12(3Δ)_*atrA/alfCD*	OP12 3Δ	PamyB:terR_ptrA; PterA:atrA_URA, PterA:alfC_argB, PterA:alfD_paba	**15**	This study
OP12(3Δ)_*atrA/alfCD pyrG⁻*	OP12(3 Δ)_*atrA/alfCD*	PamyB:terR_ptrA; PterA:atrA_URA, PterA:alfC_argB, PterA:alfD_paba *pyrG⁻*	**15**	This study
OP12(3Δ)_*atrA/alfCDB*	OP12(3Δ)_*atrA/alfCD pyrG⁻*	PamyB:terR_ptrA; PterA:atrA_URA, PterA:alfC_URA, PterA:alfD+PtrpC-alfB_URA	**15**	This study

Table S. 2: Oligonucleotides used in this study

Oligo	Sequence	Purpose
oCW77	CATTTAACAAACTTCTCATCACAGCACCATGGAGCCCAAGAATCTTTAT	amplification of alfA
oCW78	CGGTTCAGATTGAAATCACTGCTGCTCACAACCCCCGATCTTG	
oCW88	CATTTAACAAACTTCTCATCACAGCACCATGGCTACTCTCAATGAACTG	amplification of alfD
oCW89	ACGGTTCAGATTGAAATCACTGCTGCTTATGCCTTTACAACCTCAA	
oCW100	CATTTAACAAACTTCTCATCACAGCACCATGGGTTTCTTCAAGCTTGCG	amplification of alfC
oCW192	CTATACGGTTCAGATTGAAATCACTGCTGCTTATTTACTCTCATAGGGCA	
oCW190	CATTTAACAAACTTCTCATCACAGCACCATGACGTCAAAAGGCCATGTA	amplification of alfB
oCW191	CTATACGGTTCAGATTGAAATCACTGCTGCTTACCAAACATATGAACTCA	
oCW91	CATTTAACAAACTTCTCATCACAGCACCATGTCTTTCAAGAACCTCCAA	amplification of atrA
oCW92	ACGGTTCAGATTGAAATCACTGCTGCCTAAATTCCCCGTGCTTC	
oCW213	CATTTAACAAACTTCTCATCACAGCACCATGCATTATTCTCCTCTCACAG	amplification of AsMO6277
oCW214	CTATACGGTTCAGATTGAAATCACTGCTGCTCACTTAAAAATCACTCTGA	

Supplementary Figures

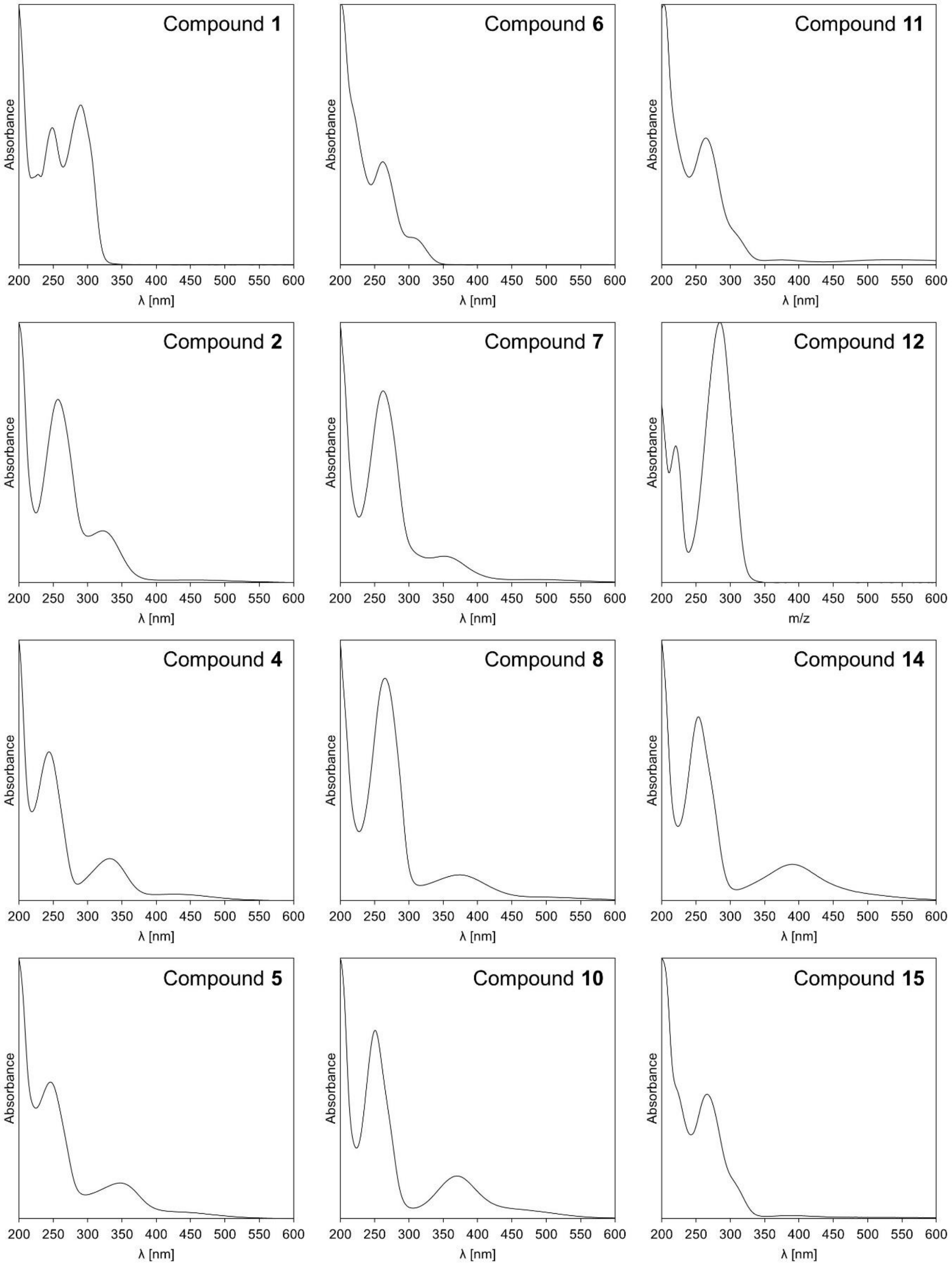

Figure S. 1: UV/Vis-spectra of reported compounds

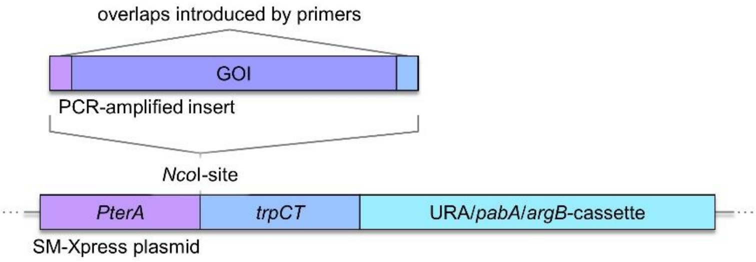

Figure S. 2: Schematic representation of strategy employed for cloning expression plasmids

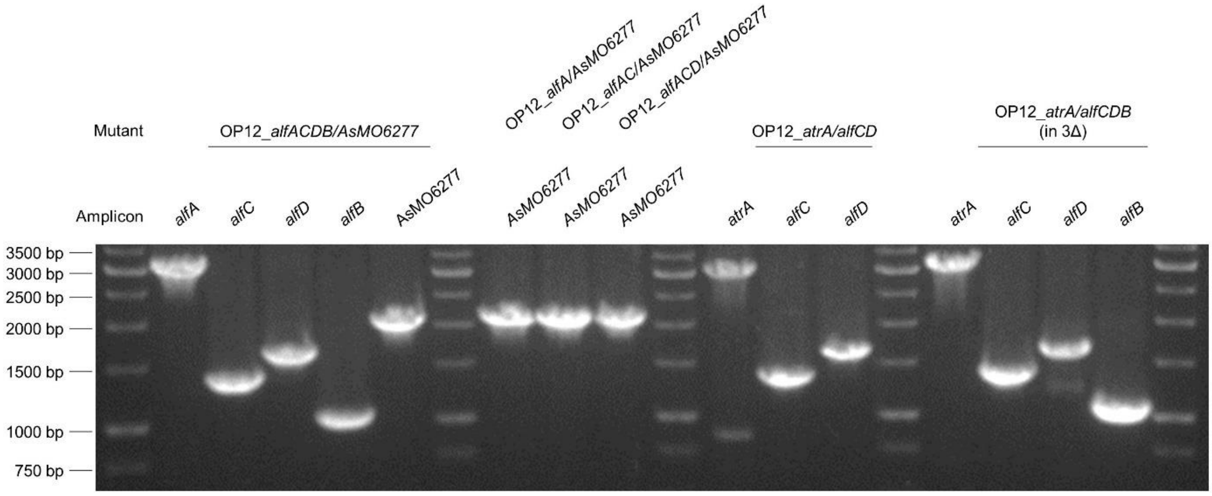

Figure S. 3: Validation of mutant strains. Genomic integration of genes was assessed by diagnostic PCR. Primers and expected amplicon sizes were as follows: *alfA*, oCW75+oCW78, 2993 bp amplicon; *alfC*, oCW75+oCW192, 1368 bp amplicon; *alfD*, oCW75+oCW89, 1632 bp amplicon; *alfB*, oCW75+oCW191, 1042 bp amplicon; *AsMO6277*, oCW75+oCW214, 2075 bp amplicon; *atrA*, oCW75+oCW92, 2964 bp amplicon.

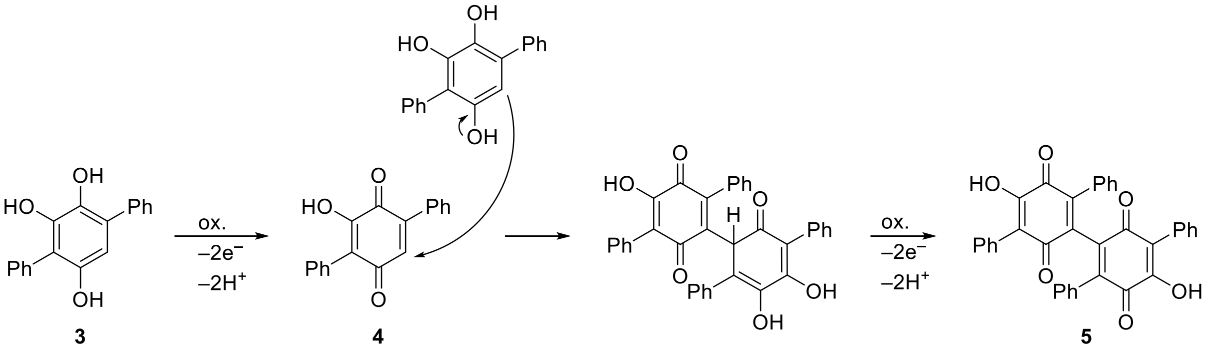

Figure S. 4: Proposed mechanism for formation of compounds **4** and **5** from compound **3**. Based on Hajdok *et al.*[3]

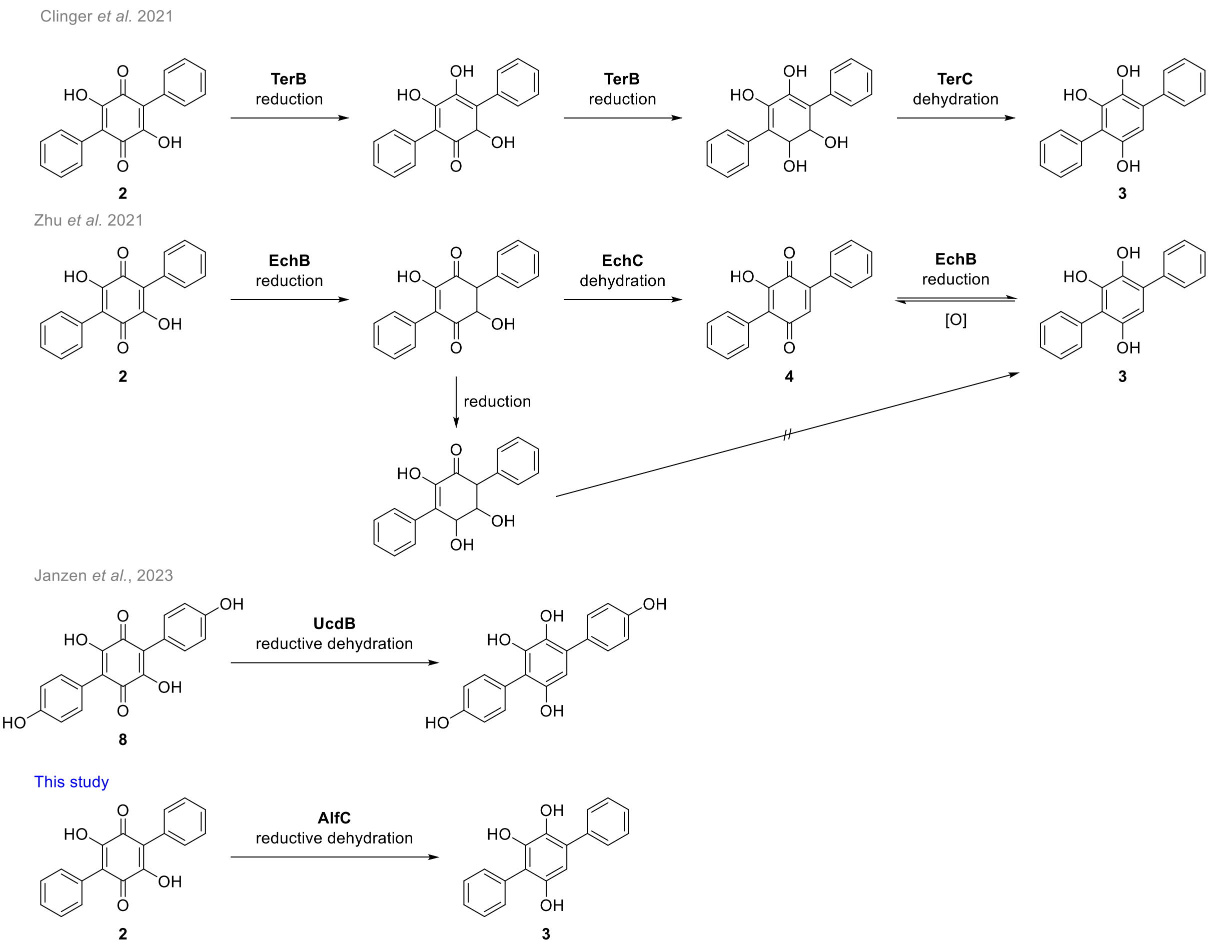

Figure S. 5:Biosynthesis of *p*-terphenyls from benzoquinones in bacteria and fungi[4–6]. Two different routes to **3** have been proposed for the biosynthesis of bacterial echosides.

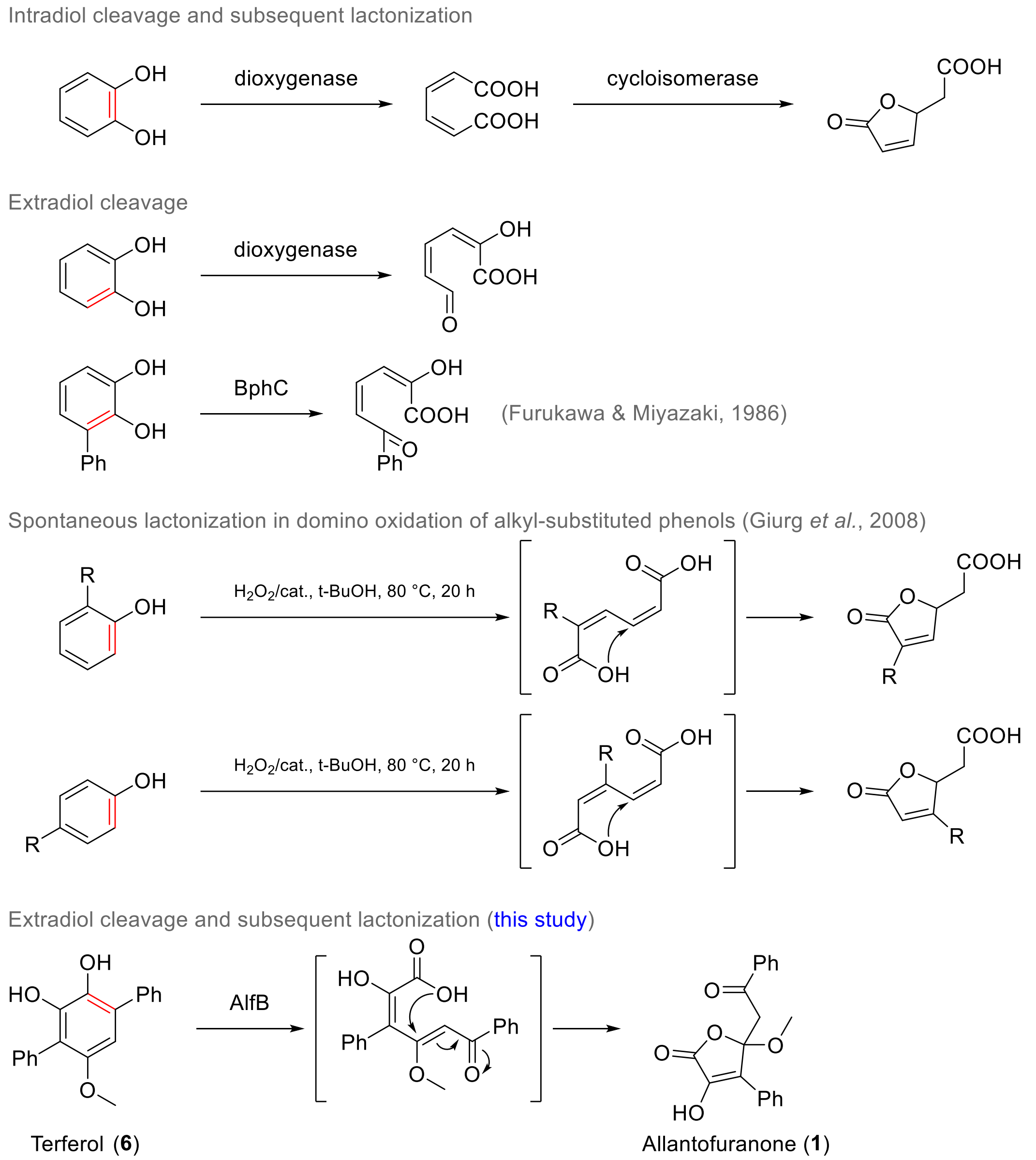

Figure S. 6: Comparison of catechol extra- and intadiol cleavage and different routes to muconolactones[7,8]

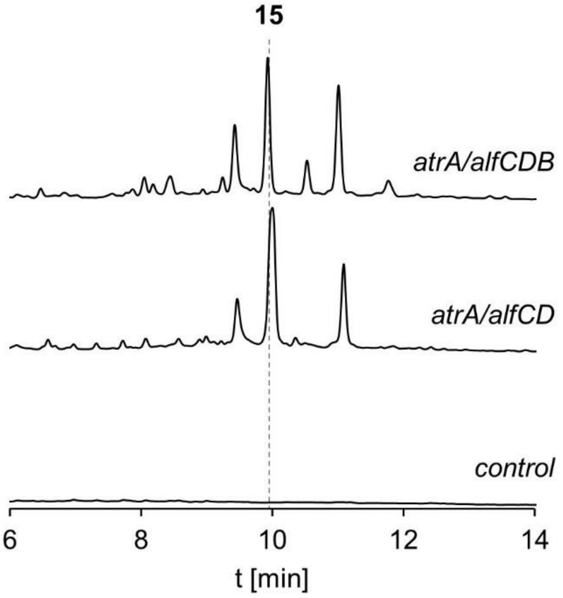

Figure S. 7: Coexpression of *alfB* in OP12(3Δ)_*atrA/alfCD* does not result in production of a dihydroxylated allantofuranone (**1**) analogue. Chromatograms (250 nm) of culture filtrate extracts of OP12 mutant strains. control, OP12 3Δ transformed with empty plasmids

NMR Assignments

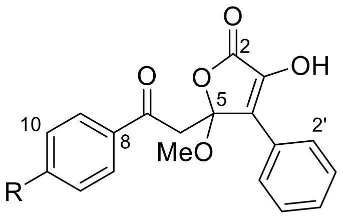

	R	
**1**	H	Allantofuranone
**12**	OH	Hydroxyallantofuranone

Table S. 3: NMR Spectroscopic Data (600 MHz) for Allantofuranone (**1**, $CDCl_3$) and Hydroxyallantofuranone (**12**, DMSO-d_6)

	**Allantofuranone (1)**		**Hydroxyallantofuranone (12)**	
**Position**	**δ_C, type**	**δ_H (*J* in Hz)**	**δ_C, type**	**δ_H (*J* in Hz)**
2	167.4, C		166.3, C	
3	140.1, C		142.1, C	
4	122.9, C		121.3, C	
5	107.5, C		106.3, C	
6	43.8, CH_2	3.90 (d, 16.5)<br>3.67 (d, 16.5)	42.9, CH_2	3.76 (d, 16.4)<br>3.68 (d, 16.4)
7	193.8, C		192.3, C	
8	136.8, C		128.3, C	
9/13	128.2, CH	7.83 (m)	130.7, CH	7.72 (m)
10/12	128.6, CH	7.40 (m)	115.0, CH	6.75 (m)
11	133.4, CH	7.53 (m)	162.2, C	
1'	129.0, C		129.8, C	
2'/6'	127.6, CH	7.89 (m)	126.7, CH	7.83 (m)
3'/5'	129.1, CH	7.44 (m)	128.7, CH	7.43 (m)
4'	129.3, CH	7.36 (m)	128.2, CH	7.33 (m)
3-OH				11.43 (br s)*
11-OH				10.41 (br s)*

* may be interchanged

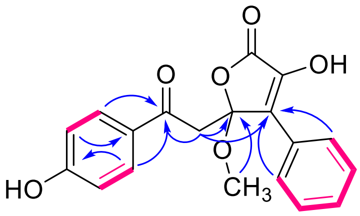

Figure S. 8: Relevant HMBC (→) and COSY (↔) correlations for **12**.

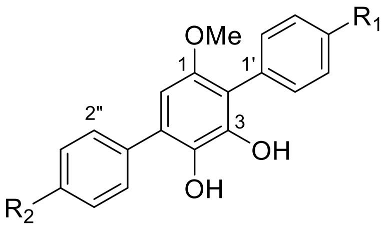

	R_1	R_2	
**6**	H	H	Terferol
**11**	H	OH	Hydroxyterferol
**15**	OH	OH	Dihydroxyterferol

Table S. 4: NMR Spectroscopic Data (600 MHz, DMSO-d_6) for Terferol (**6**), Hydroxyterferol (**11**), and Dihydroxyterferol (**15**)

	**Terferol (6)**		**Hydroxyterferol (11)**		**Dihydroxyterferol (15)**	
**Position**	**δ_C, type**	**δ_H (*J* in Hz)**	**δ_C, type**	**δ_H (*J* in Hz)**	**δ_C, type**	**δ_H (*J* in Hz)**
1	150.3, C		150.1, C		150.3, C	
2	117.8, C		117.0, C		117.0, C	
3	145.1, C		145.0, C		144.9, C	
4	136.2, C		136.0, C		136.1, C	
5	128.6, C		128.6, C		127.8, C	
6	103.5, CH	6.44 (s)	103.0, CH	6.38 (s)	103.0, CH	6.35 (s)
1'	134.4, C		134.5, C		124.7, C	
2'/6'	130.8, CH	7.30 (m)	130.8, CH	7.28 (m)	131.8, CH	7.08 (m)
3'/5'	127.4, CH	7.37 (m)	127.4, CH	7.36 (m)	114.3, CH	6.75 (m)
4'	126.3, CH	7.28 (m)	126.8, CH	7.26 (m)	155.8, C	
1"	138.9, C		129.4, C		129.5, C	
2"/6"	129.0, CH	7.61 (m)	130.1, CH	7.43 (m)	130.0, CH	7.42 (m)
3"/5"	128.0, CH	7.43 (m)	114.8, CH	6.82 (m)	114.7, CH	6.80 (m)
4"	126.6, CH	7.33 (m)	156.3, C		156.2, C	
1-OMe	55.6, CH_3	3.62 (s)	55.5, CH_3	3.60 (s)	55.5, CH_3	3.59 (s)
3-OH		8.33 (s)		8.23 (s)		8.17 (br s)
4-OH		8.03 (s)		7.90 (s)		7.91 (br s)
4'-OH						9.33 (br s)
4"-OH				9.45 (s)		9.44 (s)

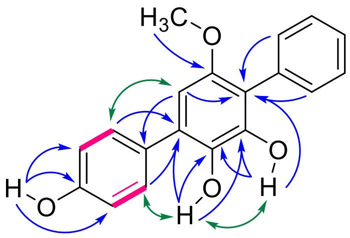

Figure S. 9: Relevant HMBC (→), COSY (↔), and NOESY (↔) correlations for **11**.

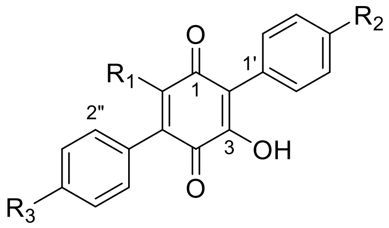

	R_1	R_2	R_3	
**2**	OH	H	H	Polyporic acid
**4**	H	H	H	Deoxypolyporic acid
**5**	Dimer	H	H	Deoxypolyporic acid dimer
**7**	OH	OH	H	Ascocorynin
**8**	OH	OH	OH	Atromentin
**10**	H	OH	H	Deoxyascocorynin

Table S. 5: NMR Spectroscopic Data (600 MHz, DMSO-d_6) for Polyporic acid (**2**), Deoxypolyporic acid (**4**), Deoxypolyporic acid dimer (**5**), Ascocorynin (**7**), Atromentin (**8**), Deoxyascocorynin (**10**)

	**Polyporic acid (2)**		**Deoxypolyporic acid (4)**		**Deoxypolyporic acid dimer (5)**		**Ascocorynin (7)**		**Atromentin (8)**		**Deoxyascocorynin (10)**	
**Position**	**δ_C, type**	**δ_H (*J* in Hz)**	**δ_C, type**	**δ_H (*J* in Hz)**	**δ_C, type**	**δ_H (*J* in Hz)**	**δ_C, type**	**δ_H (*J* in Hz)**	**δ_C, type**	**δ_H (*J* in Hz)**	**δ_C, type**	**δ_H (*J* in Hz)**
1	168.0, C[a]		182.7, C		182.0, C		168.2, C[a]		168.3, C[a]		183.3 C	
2	115.4, C		118.7, C		119.3, C		115.5, C		114.5, C		118.4, C	
3	168.0, C[a]		153.4, C		152.8, C		168.2, C[a]		168.3, C[a]		153.3, C	
4	168.0, C[a]		186.2, C		185.2, C		168.2, C[a]		168.3, C[a]		186.2, C	
5	115.4, C		142.2, C		140.4, C		115.2, C		114.5, C		141.7, C	
6	168.0, C[a]		133.4, CH	6.88 (s)	140.0, C		168.2, C[a]		168.3, C[a]		131.2, CH	6.78 (s)
1'	130.8, C		131.0, C		131.2, C		120.9, C		121.6, C		131.1, C	
2'/6'	130.4, CH	7.39 (m)	130.5, CH	7.35 (m)	130.5, CH	7.27 (m)	131.6, CH	7.22 (m)	131.6, CH	7.23 (m)	130.5, CH	7.34 (m)
3'/5'	127.5, CH	7.41 (m)	127.4, CH	7.40 (m)	127.8, CH	7.34 (m)	114.4, CH	6.78 (m)	114.2, CH	6.76 (m)	127.3, CH	7.38 (m)
4'	127.4, CH	7.33 (m)	127.4, CH	7.34 (m)	127.7, CH	7.34 (m)	156.7, C		156.4, C		127.3, CH	7.32 (m)
1"	130.8, C		132.5, C		130.6, C		130.8, C		121.6, C		123.1, C	
2"/6"	130.4, CH	7.39 (m)	129.1, CH	7.59 (m)	128.9, CH	6.75 (m)	130.4, CH	7.37 (m)	131.6, CH	7.23 (m)	130.7, CH	7.47 (m)
3"/5"	127.5, CH	7.41 (m)	128.2, CH	7.48 (m)	127.5, CH	7.39 (m)	127.5, CH	7.40 (m)	114.2, CH	6.76 (m)	115.2, CH	6.85 (m)
4"	127.4, CH	7.33 (m)	129.5, CH	7.48 (m)	128.9, CH	7.39 (m)	127.3, CH	7.32 (m)	156.4, C		159.0, C	
3-OH (6-OH)		11.17 (br s)		10.99 (br s)		11.27 (br s)		10.99 (br s)		10.73 (br s)		10.85 (br s)
4'-OH (4"-OH)								9.56 (s)		9.49 (br s)		9.93 (s)

[a] very broad

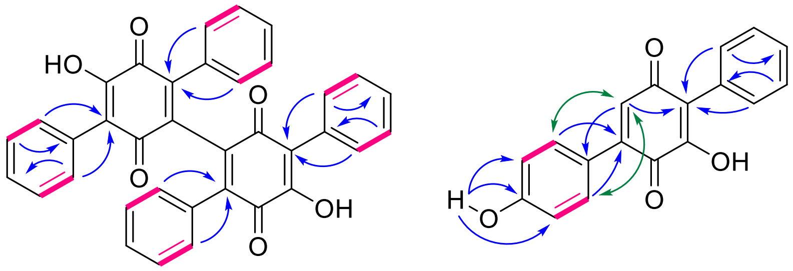

5 **10**

Figure S. 11: Relevant HMBC (→), COSY (↔), and NOESY (↔) correlations for **5** and **10**.

NMR Spectra

NMR spectra (1D and 2D), raw data, and additional analytical data of all compounds can be found free of charge on Chemotion Repository (Table S. 3). Additionally, ^{1}H and ^{13}C NMR spectra of the new compounds are depicted on the following pages.

Table S. 6: Deposited analytical data

	Name	DOI
**1**	Allantofuranone	https://dx.doi.org/10.14272/ZSQDINYGPVLTCM-UHFFFAOYSA-N.1
**2**	Polyporic acid	https://dx.doi.org/10.14272/HZKFHDXTSAYOSN-UHFFFAOYSA-N.1
**4**	Deoxypolyporic acid	https://dx.doi.org/10.14272/GXMJKXBNCXNACS-UHFFFAOYSA-N.1
**5**	Deoxypolyporic acid dimer	https://dx.doi.org/10.14272/JIJBMATWFKIPNS-UHFFFAOYSA-N.1
**6**	Terferol	https://dx.doi.org/10.14272/HMCUGCPHZLJKMS-UHFFFAOYSA-N.1
**7**	Ascocorynin	https://dx.doi.org/10.14272/PNTORJXTFRBTDZ-UHFFFAOYSA-N.1
**8**	Atromentin	https://dx.doi.org/10.14272/FKQQKMGWCJGUCS-UHFFFAOYSA-N.1
**10**	Deoxyascocorynin	https://dx.doi.org/10.14272/HLFCRNGNTFLPPX-UHFFFAOYSA-N.1
**11**	Hydroxyterferfol	https://dx.doi.org/10.14272/NJXLDQMVOFLMIX-UHFFFAOYSA-N.1
**12**	Hydroxyallantofuranone	https://dx.doi.org/10.14272/LQKBSBWZJOHANT-UHFFFAOYSA-N.1
**15**	Dihydroxyterferol	https://dx.doi.org/10.14272/HDXIDAAYQIUQQS-UHFFFAOYSA-N.1

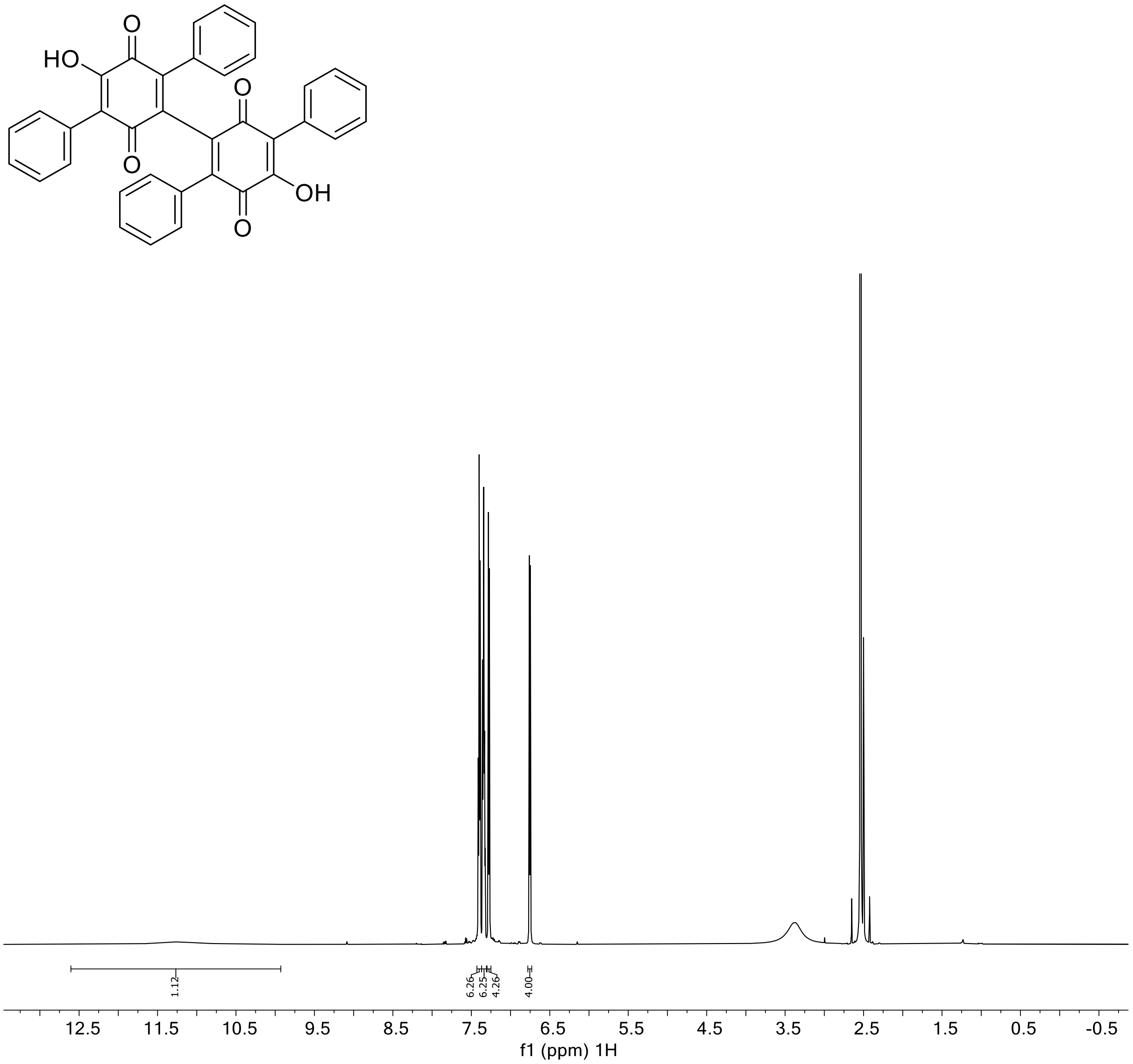

Spectrum S. 1: 1H NMR spectrum (600 MHz, DMSO-d_6) of the new compound deoxypolyporic acid dimer (**5**)

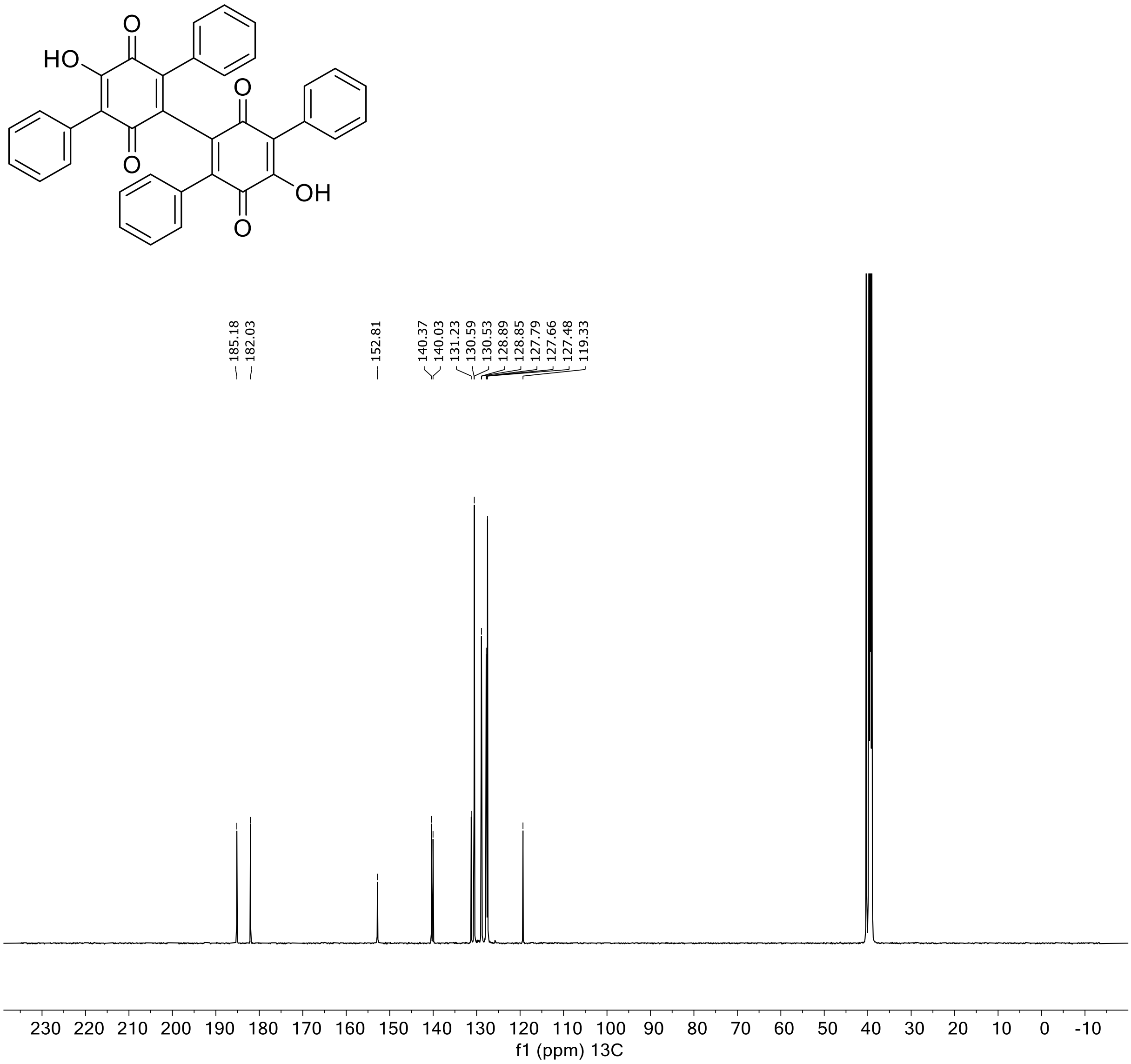

Spectrum S. 2: 13C NMR spectrum (600 MHz, DMSO-d_6) of the new compound deoxypolyporic acid dimer (**5**)

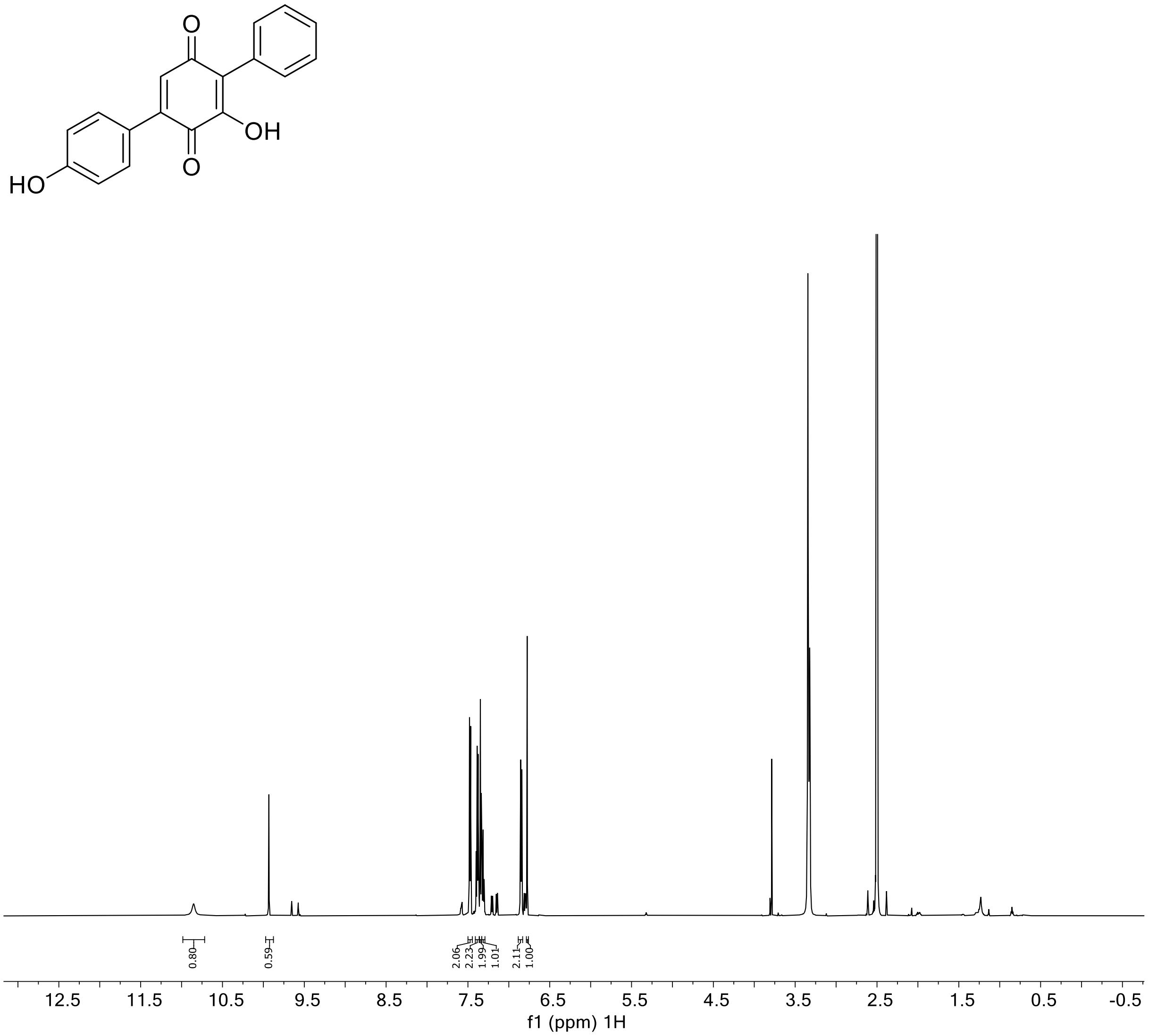

Spectrum S. 3: 1H NMR spectrum (600 MHz, DMSO-d_6) of the new compound deoxyascocorynin (**10**)

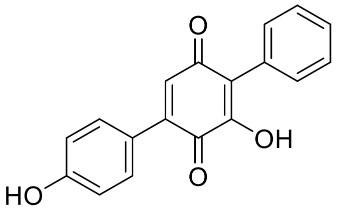

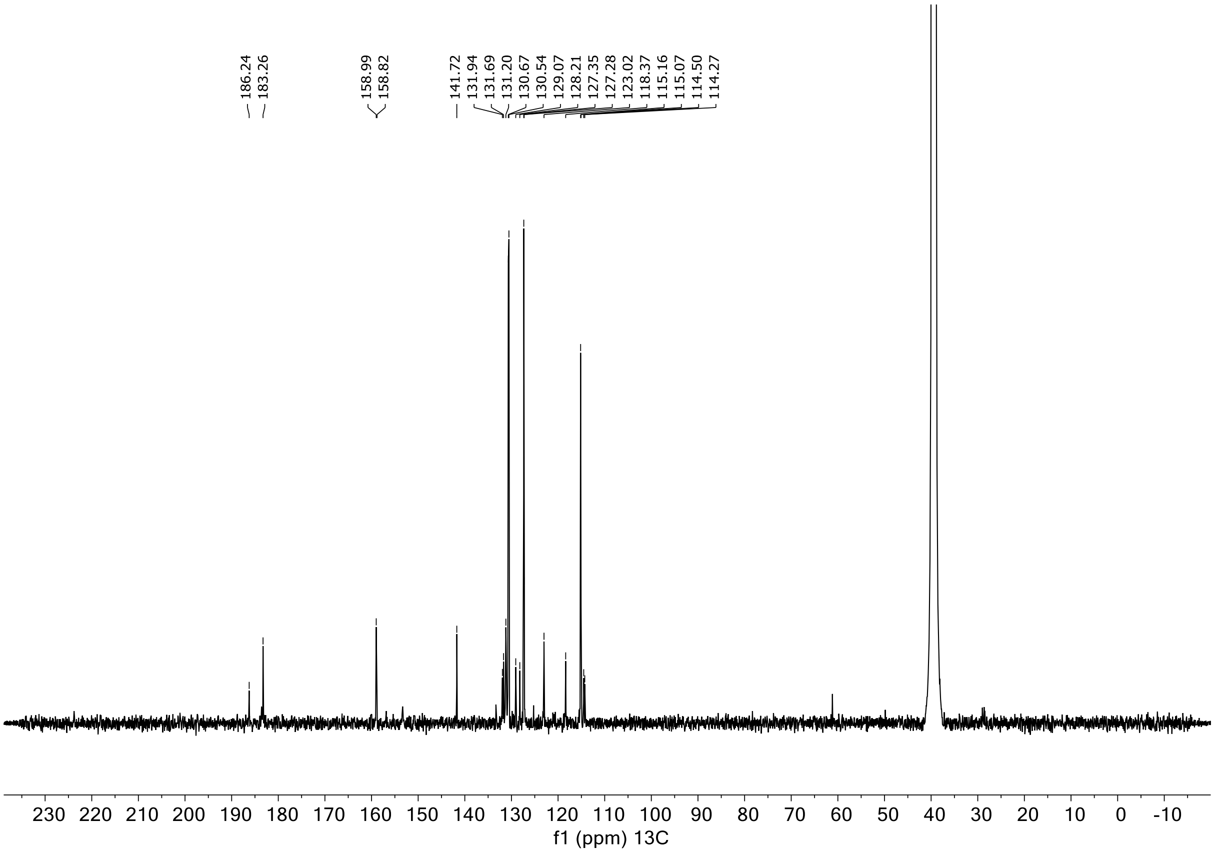

Spectrum S. 4: 13C NMR spectrum (600 MHz, DMSO-d_6) of the new compound deoxyascocorynin (**10**)

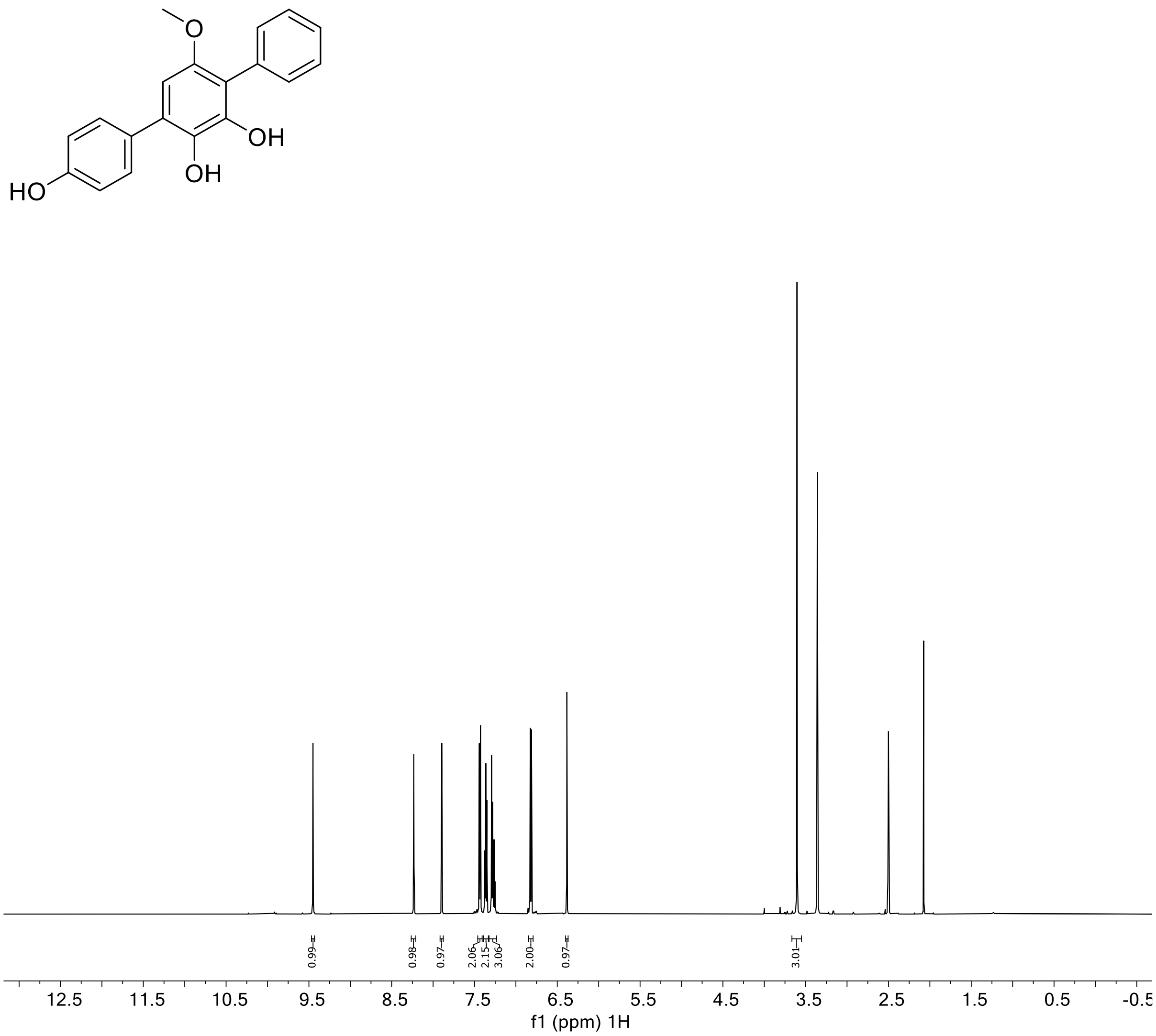

Spectrum S. 5: 1H NMR spectrum (600 MHz, DMSO-d_6) of the new compound hydroxyterferol (**11**)

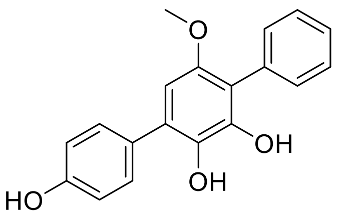

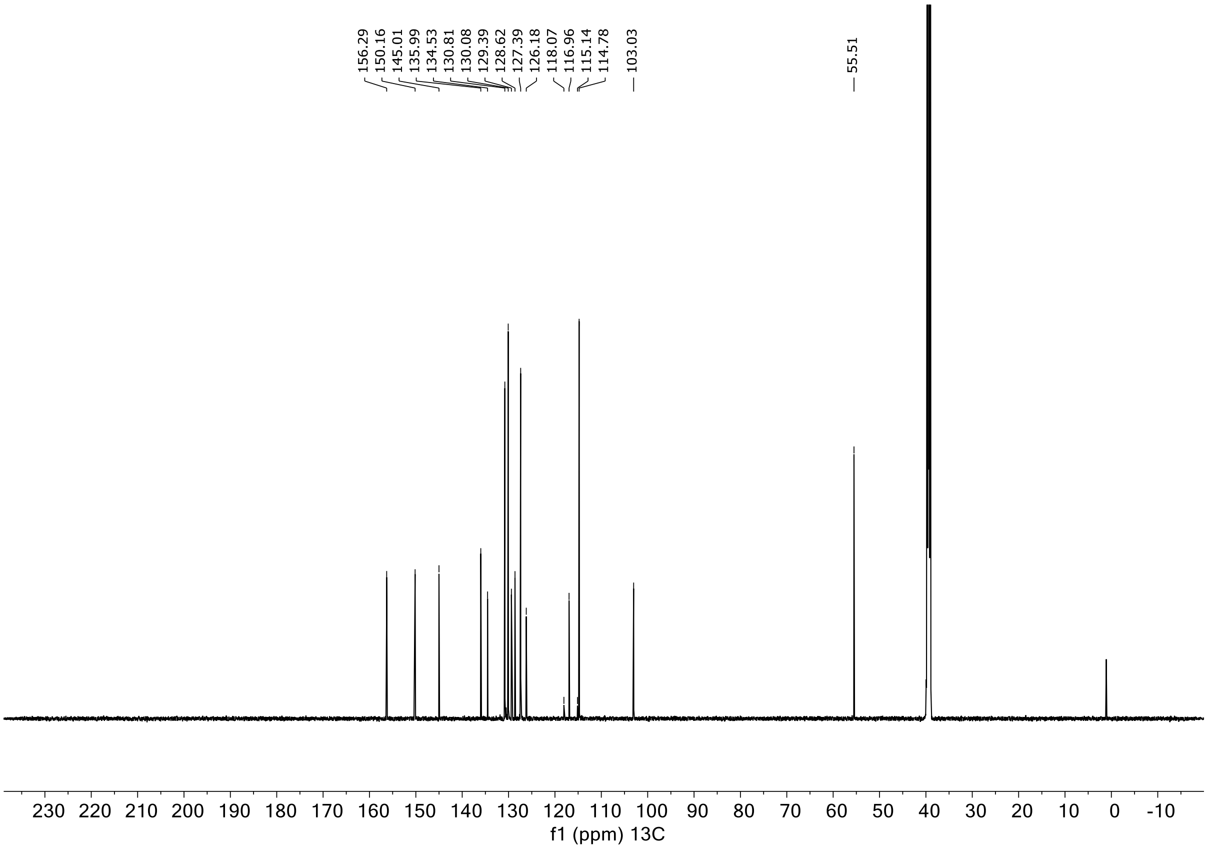

Spectrum S. 6: 13C NMR spectrum (600 MHz, DMSO-d_6) of the new compound hydroxyterferol (**11**)

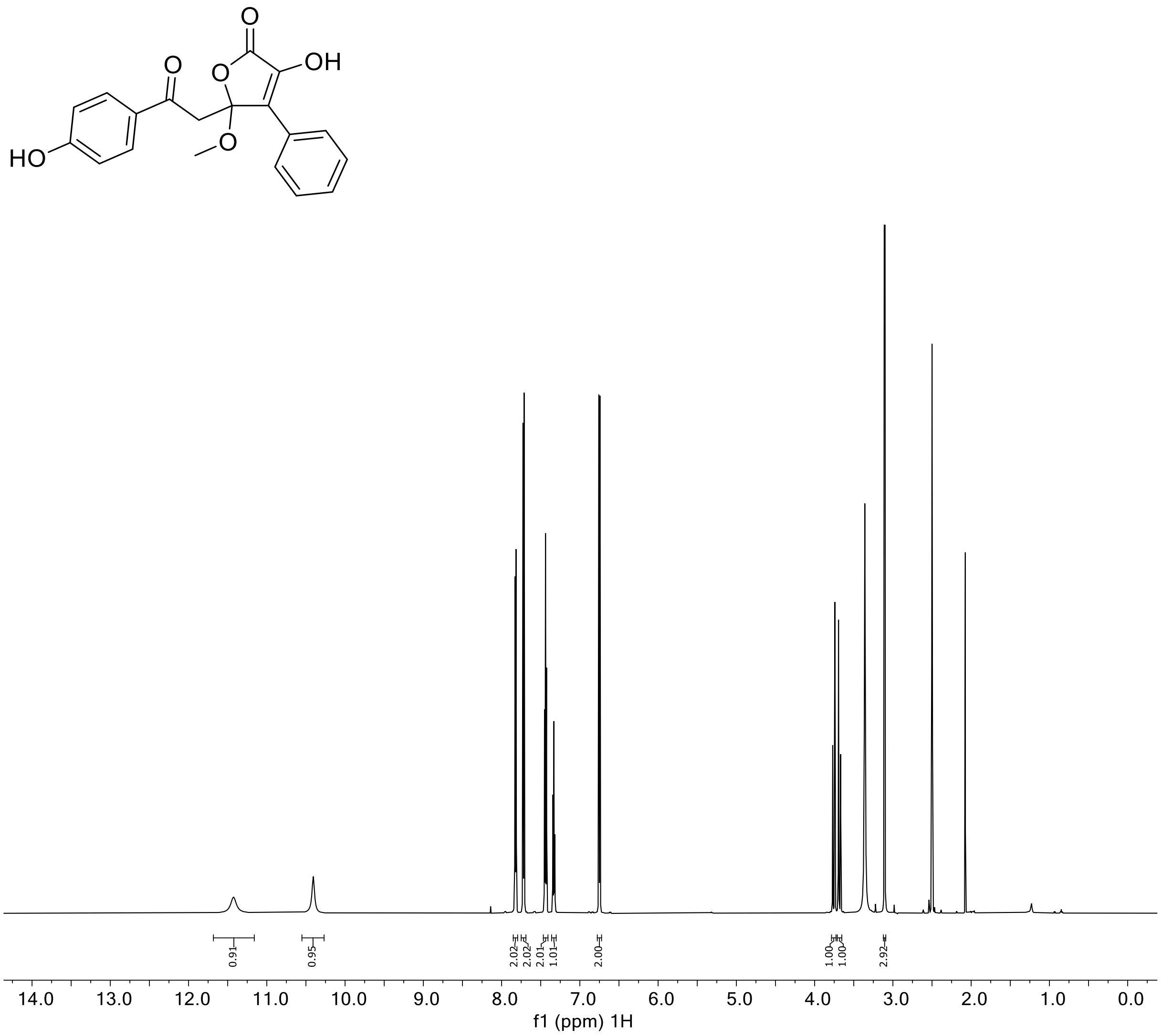

Spectrum S. 7: 1H NMR spectrum (600 MHz, DMSO-d_6) of the new compound hydroxyallantofuranone (**12**)

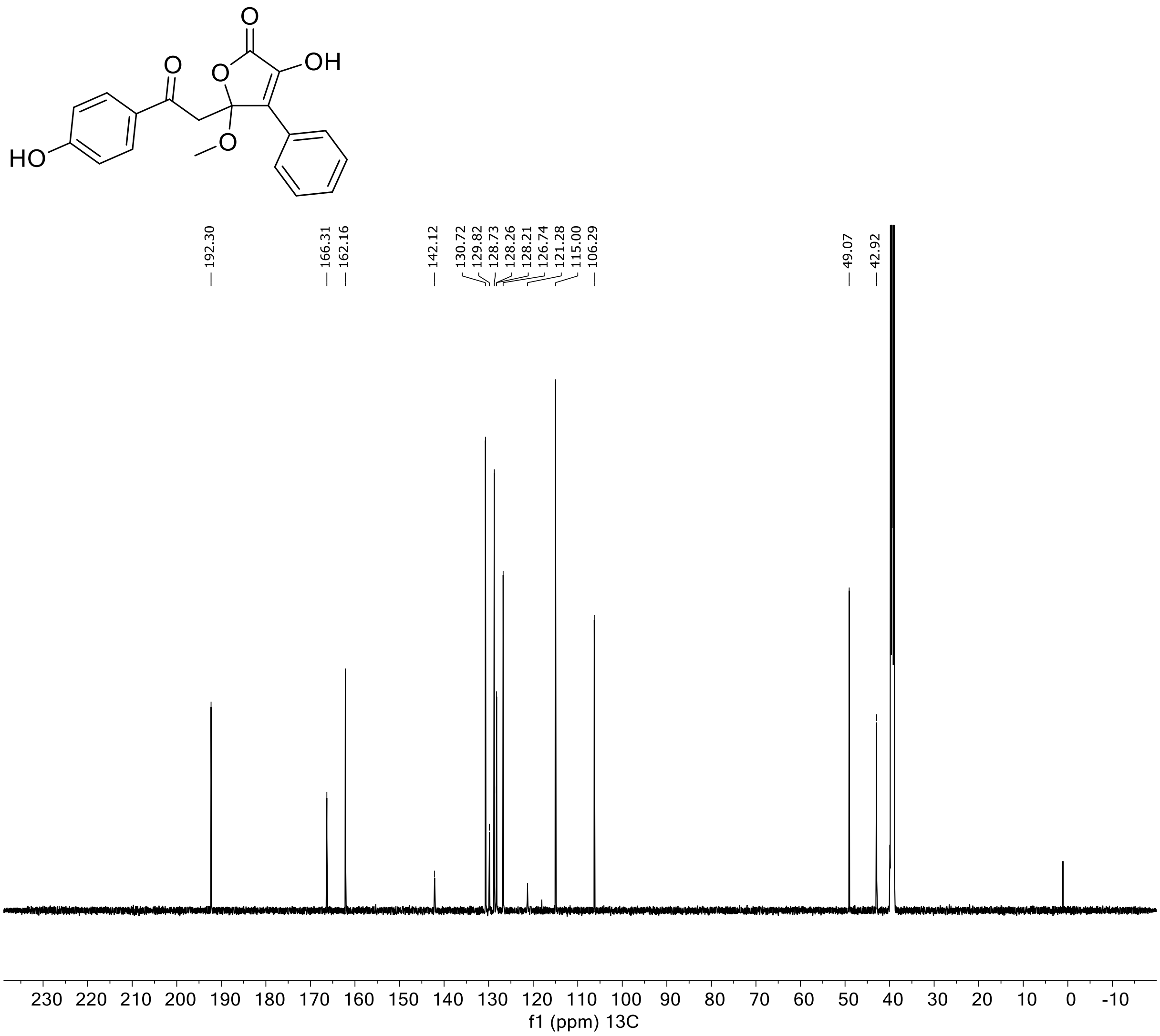

Spectrum S. 8: 13C NMR spectrum (600 MHz, DMSO-d_6) of the new compound hydroxyallantofuranone (**12**)

References

(1) Geib, E.; Baldeweg, F.; Doerfer, M.; Nett, M.; Brock, M. Cross-Chemistry Leads to Product Diversity from Atromentin Synthetases in Aspergilli from Section Nigri. *Cell chemical biology* **2019**, *26* (2), 223-234.e6. DOI: 10.1016/j.chembiol.2018.10.021.

(2) Wieder, C.; Künzer, M.; Wiechert, R.; Seipp, K.; Andresen, K.; Stark, P.; Schüffler, A.; Opatz, T.; Thines, E. Biosynthesis of the Antifungal Polyhydroxy-Polyketide Acrophialocinol. *Organic letters* **2025**, *27* (4), 1036–1041. DOI: 10.1021/acs.orglett.4c04656.

(3) Hajdok, S.; Conrad, J.; Beifuss, U. Laccase-catalyzed domino reactions between hydroquinones and cyclic 1,3-dicarbonyls for the regioselective synthesis of substituted p-benzoquinones. *The Journal of organic chemistry* **2012**, *77* (1), 445–459. DOI: 10.1021/jo202082v.

(4) Clinger, J. A.; Zhang, Y.; Liu, Y.; Miller, M. D.; Hall, R. E.; van Lanen, S. G.; Phillips, G. N.; Thorson, J. S.; Elshahawi, S. I. Structure and Function of a Dual Reductase-Dehydratase Enzyme System Involved in p-Terphenyl Biosynthesis. *ACS chemical biology* **2021**, *16* (12), 2816–2824. DOI: 10.1021/acschembio.1c00701.

(5) Zhu, J.; Liu, M.; Deng, J.; Chen, W.; Zhu, D.; Duan, J.; Li, Y.; Wang, H.; Shen, Y. The coupled reaction catalyzed by EchB and EchC lead to the formation of the common 2',3',5'-trihydroxy-benzene core in echosides biosynthesis. *Biochemical and biophysical research communications* **2021**, *559*, 62–69. DOI: 10.1016/j.bbrc.2021.04.087.

(6) Janzen, D. J.; Zhou, J.; Li, S.-M. Biosynthesis of p-Terphenyls in Aspergillus ustus Implies Enzymatic Reductive Dehydration and Spontaneous Dibenzofuran Formation. *Organic letters* **2023**, *25* (34), 6311–6316. DOI: 10.1021/acs.orglett.3c02234.

(7) Furukawa, K.; Miyazaki, T. Cloning of a gene cluster encoding biphenyl and chlorobiphenyl degradation in Pseudomonas pseudoalcaligenes. *Journal of bacteriology* **1986**, *166* (2), 392–398. DOI: 10.1128/jb.166.2.392-398.1986.

(8) Giurg, M.; Kowal, E.; Muchalski, H.; Syper, L.; Młochowski, J. Catalytic Oxidative Domino Degradation of Alkyl Phenols Towards 2- and 3-Substituted Muconolactones. *Synthetic Communications* **2008**, *39* (2), 251–266. DOI: 10.1080/00397910802369687.
